# Nanovaccine-based strategies for lymph node targeted delivery and imaging in tumor immunotherapy

**DOI:** 10.1186/s12951-023-01989-x

**Published:** 2023-07-23

**Authors:** Ao He, Xiaoye Li, Zhuo Dai, Qiang Li, Yu Zhang, Meng Ding, Zhi-fa Wen, Yongbin Mou, Heng Dong

**Affiliations:** 1 Nanjing Stomatological Hospital, Affiliated Hospital of Medical School, Nanjing University, 30 Zhongyang Road, Nanjing, 210008 China; 2 Department of Clinical Laboratory, Women’s Hospital of Nanjing Medical University, Nanjing Maternity and Child Health Care Hospital, Nanjing, 210004 China

**Keywords:** Nanovaccines, Tumor vaccines, Targeting lymph node, Lymph node imaging, Tumor immunotherapies

## Abstract

Therapeutic tumor vaccines have attracted considerable attention in the past decade; they can induce tumor regression, eradicate minimal residual disease, establish lasting immune memory and avoid non-specific and adverse side effects. However, the challenge in the field of therapeutic tumor vaccines is ensuring the delivery of immune components to the lymph nodes (LNs) to activate immune cells. The clinical response rate of traditional therapeutic tumor vaccines falls short of expectations due to inadequate lymph node delivery. With the rapid development of nanotechnology, a large number of nanoplatform-based LN-targeting nanovaccines have been exploited for optimizing tumor immunotherapies. In addition, some nanovaccines possess non-invasive visualization performance, which is benefit for understanding the kinetics of nanovaccine exposure in LNs. Herein, we present the parameters of nanoplatforms, such as size, surface modification, shape, and deformability, which affect the LN-targeting functions of nanovaccines. The recent advances in nanoplatforms with different components promoting LN-targeting are also summarized. Furthermore, emerging LNs-targeting nanoplatform-mediated imaging strategies to both improve targeting performance and enhance the quality of LN imaging are discussed. Finally, we summarize the prospects and challenges of nanoplatform-based LN-targeting and /or imaging strategies, which optimize the clinical efficacy of nanovaccines in tumor immunotherapies.

## Introduction

With rapid advances in medical immunology, tumor immunotherapies, such as chimeric antigen receptor T cells (CAR-T), immune checkpoint blockers (ICBs) and tumor vaccines, have become effective treatment methods [1–3]. Their common goals are to treat tumors by enhancing antitumor immune responses, reprogramming the tumor microenvironment (TME) and inhibiting the malignant growth of tumors [4]. Among them, tumor vaccines have received increasing attention because they can deliver tumor antigens and adjuvants to activate antigen-presenting cells (APCs) and then trigger a robust antitumor immune response with long-term immune memory, which has shown significant therapeutic effects on suppressing tumor growth, recurrence and metastasis [5]. However, the clinical implementation of tumor vaccines is still limited mainly due to inadequate targeted delivery of vaccine components to lymph nodes (LNs) [3, 6, 7]. The LN is a second lymphoid organ where mature lymphocytes (T cells and B cells) reside, and it is the main site where lymphocytes generate an immune response to foreign antigens [8]. Thus, the LN as a target site for the delivery of antigens and adjuvants is essential for the role of tumor vaccines. The most important rate-limiting step for immune induction is the ability of tumor vaccines to target LNs and be taken up by APCs in LNs [8]. In addition, some particular LNs, especially tumor-draining lymph nodes (TDLNs), need targeted delivery of tumor vaccines. TDLNs play a key role in the development and treatment of tumors, collecting lymphatic flow containing tumor-derived cells and factors to store tumor-specific T cells and inducing them to infiltrate and inhibit primary tumors [9]. Thus, it is important to develop more advanced LN-targeting tumor vaccines to achieve satisfactory antitumor outcomes.

With recent developments in the fields of nanotechnology and bioengineering, applications of nanoplatform-optimized tumor vaccines, such as nanovaccines, have provided a novel strategy for promoting immune agents targeting LNs [10]. The leakage of antigen or adjuvant during vaccine administration would result in weak immunogenicity. Compared with conventional tumor vaccines, nanovaccines can simultaneously package antigen and adjuvant and deliver them to APCs, which can avoid leakage of antigen or adjuvant to improve the therapeutic effect of vaccines [11–13]. LN-targeting nanovaccines can promote adaptive immune responses by enhancing the antigen uptake efficiency of APCs, especially when nanoplatforms are modified with targeted ligand [14]. These nanovaccines can target lymphoid tissues and APCs to enhance the efficacy of the lymph pump in vivo pharmacology, inducing precise immunomodulation [11, 15–17]. In addition, loading nanoplatforms with tracers for LN imaging can support understanding of the dynamics of vaccine exposure in lymphoid tissue. Meanwhile, the risk of tumor metastasis via the lymphatic system is high due to tumor dissemination. Thus, localization of LN metastasis by nanoplatforms is an appealing strategy to achieve accurate diagnosis and precision tumor treatment.

**Fig. 1:**
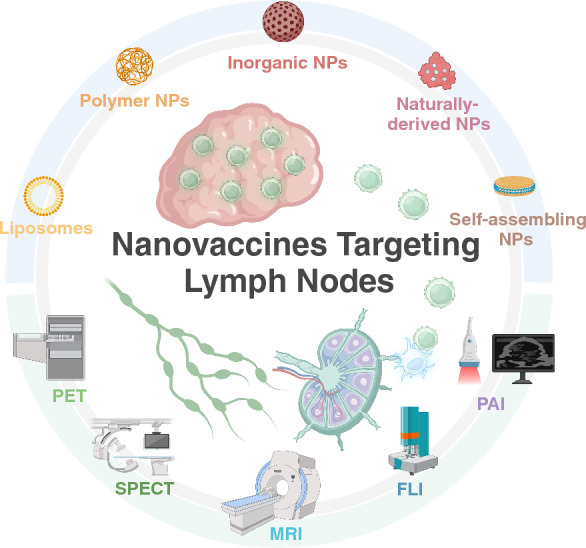
Various nanoplatforms have been applied for lymph node targeted delivery and imaging in tumor immunotherapy. Different nanoplatforms, such as liposomes, polymer nanoplatforms (NPs), inorganic NPs, naturally derived NPs, or self-assembling NPs, have been employed to promote lymph node (LN)-targeted delivery in antitumor immune responses. Moreover, nanoplatform-mediated theranostics by various imaging techniques, such as positron emission tomography (PET) and single-photon emission computed tomography (SPECT), magnetic resonance imaging (MRI), fluorescence imaging (FLI) and photoacoustic imaging (PAI), can synergistically promote the effects of diagnosis and treatment in tumor immunotherapy

In this review, we first describe the importance of targeting LNs in tumor immunotherapy. Next, we summarize the various parameters of nanoplatforms, including size, surface modification, shape and deformability, which affect LN-targeting performance. Furthermore, the current advances of different LN-targeting nanovaccines, including lipid-based, polymeric, inorganic, naturally derived, and self-assembling nanoplatforms, are also described in studies of tumor immunotherapy. In addition, LN-targeting imaging method-based nanovaccines and their application are illustrated according to the classifications of acellular nanovaccines and nanovaccines loading autologous cells (Fig. 1). Finally, the challenges and future prospects of nanovaccines prompting LN targeting and imaging modalities are also discussed.

## The importance of targeting LNs in tumor immunotherapy

**Fig. 2:**
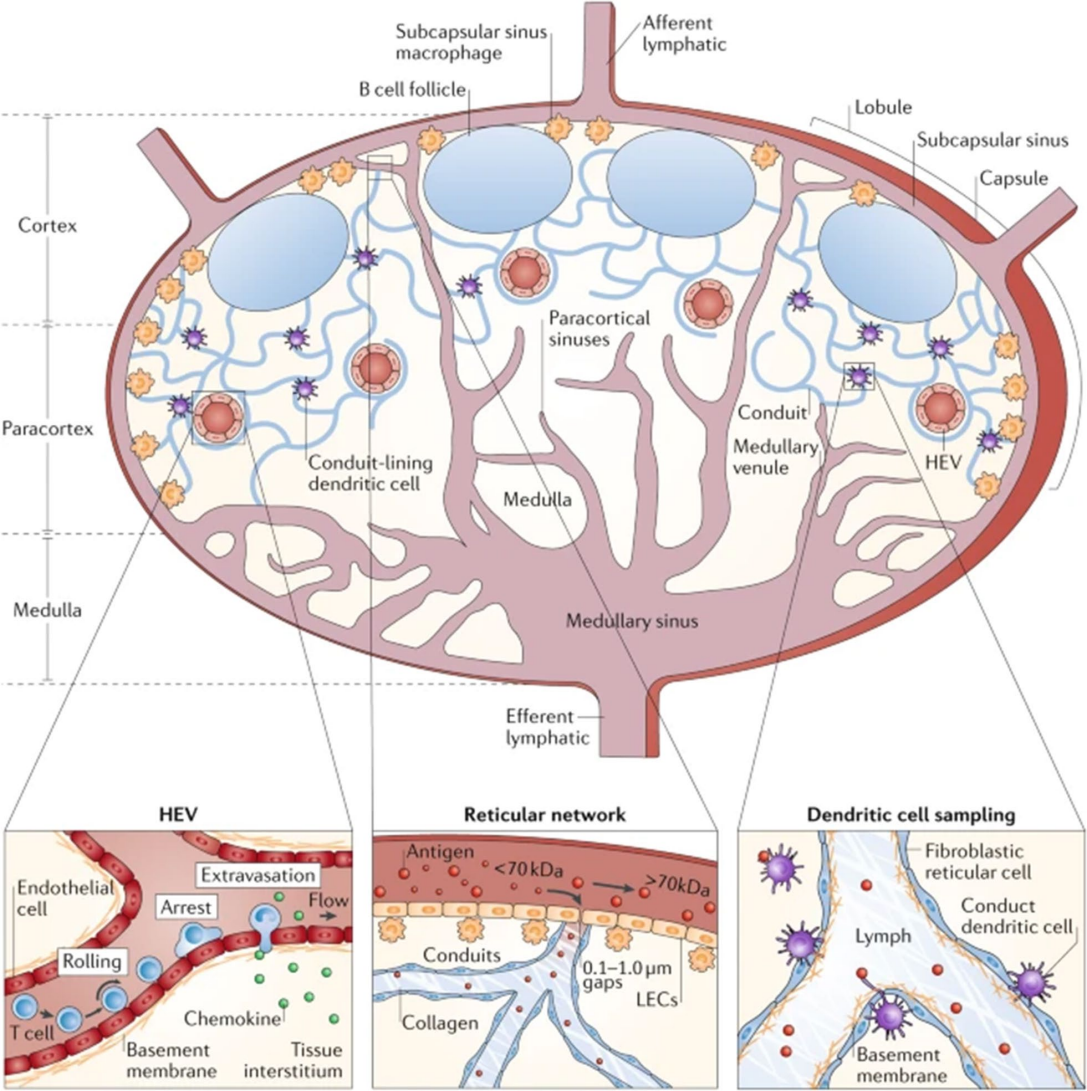
The structure and physiology of lymph nodes. The architecture of the LNs can be divided into distinct areas, including cortex, paracortex and medulla. Naïve lymphocytes enter LNs via HEVs or afferent lymphatic vessels and exit through cortical sinuses, medullary sinuses and efferent lymphatic vessels in the medulla. DCs enter the LNs via afferent lymphatic vessels and sub-peritoneal sinuses or HEVs. The cortex contains a dense population of B cells and follicular dendritic cells (FDCs) arranged in discrete B cell follicles, with T cells clustered in the T cell zones of the paracortex. Fibroblast reticulocytes (FRCs) in the paracortical T cell areas form a network of reticulocytes and stroma that act as a guidance pathway for lymphocytes and DCs (Adapted with permission from [18]. Copyright © 2012, Springer Nature Limited)

LNs are vital tissues in the immune system that provide a structure for collecting immunogenicity information from peripheral tissues and then reenter the cycle to provide protective immunity on the periphery (Fig. 2) [18]. Therefore, direct delivery of vaccines to LNs offers an opportunity to address a variety of local and systemic immune challenges.

### Anatomical physiology of LNs

LNs are located along the course of lymphatic vessels, which are the most complete peripheral immune organ and consist of fluid-filled lumen structures, cellular locations and structural units (the cortical area and the medullary area) [19]. LNs are covered with dense connective tissue, some of which extend into LNs in a trabecular fashion, forming many lacunae. The lymphoid follicle of the cortex receives lymph from afferent vessels and forms germinal centers by proliferation of B cells when stimulated by antigens [20]. T-cell settles are paracortical areas between the cortex and medulla, and APCs can present processed antigen peptides to T cells for activation and proliferation in the paracortex.

The lymphatic vessels have valves similar to veins to prevent lymph reflux [21], and the vigorous contractions of the smooth muscle cells lining the collector drive the flow away from the tissue drainage sites [22]. The structure of the subcapsular sinus (SCS) plays a key role in lymphatic drainage. The wall of the subcapsular sinus is a monolayer of discontinuous lymphatic endothelial cells (LECs) that can prevent free lymph from entering LNs [23]. Therefore, most lymph is dispersed in LNs through the sinuses and eventually passed from LNs through the efferent lymphatic vessels, while a portion of lymph and small molecules can enter the LN parenchyma through the conduit system, which is an interconnected network composed of fibroblast reticular cells [20, 24]. Another important structure of LNs is the high endothelial venule (HEV), which consists of endothelial cells in the paracortex. HEV controls the type of lymphocytes and the location where they enter lymphoid tissues while expressing vascular address proteins for regulation [25]. Meanwhile, the lymphocytes in the blood enter the substance of LNs through the HEV. Thus, the HEV is an important channel connecting the blood circulation with the lymphatic circulation, which is crucial for LN function [25, 26].

Overall, LNs and other secondary lymphatic tissues can produce highly specialized microenvironments for generating effective immune responses. The structural features of LNs make them the best site for adaptive immunity [20, 27]. Furthermore, LNs can not only remove pathogenic foreign bodies through APCs in the sinuses or migrate from peripheral tissues to effectively filter lymph but also maintain a normal immune response through lymphocyte recycling [28].

### The role of tumor-draining lymph nodes

**Fig. 3:**
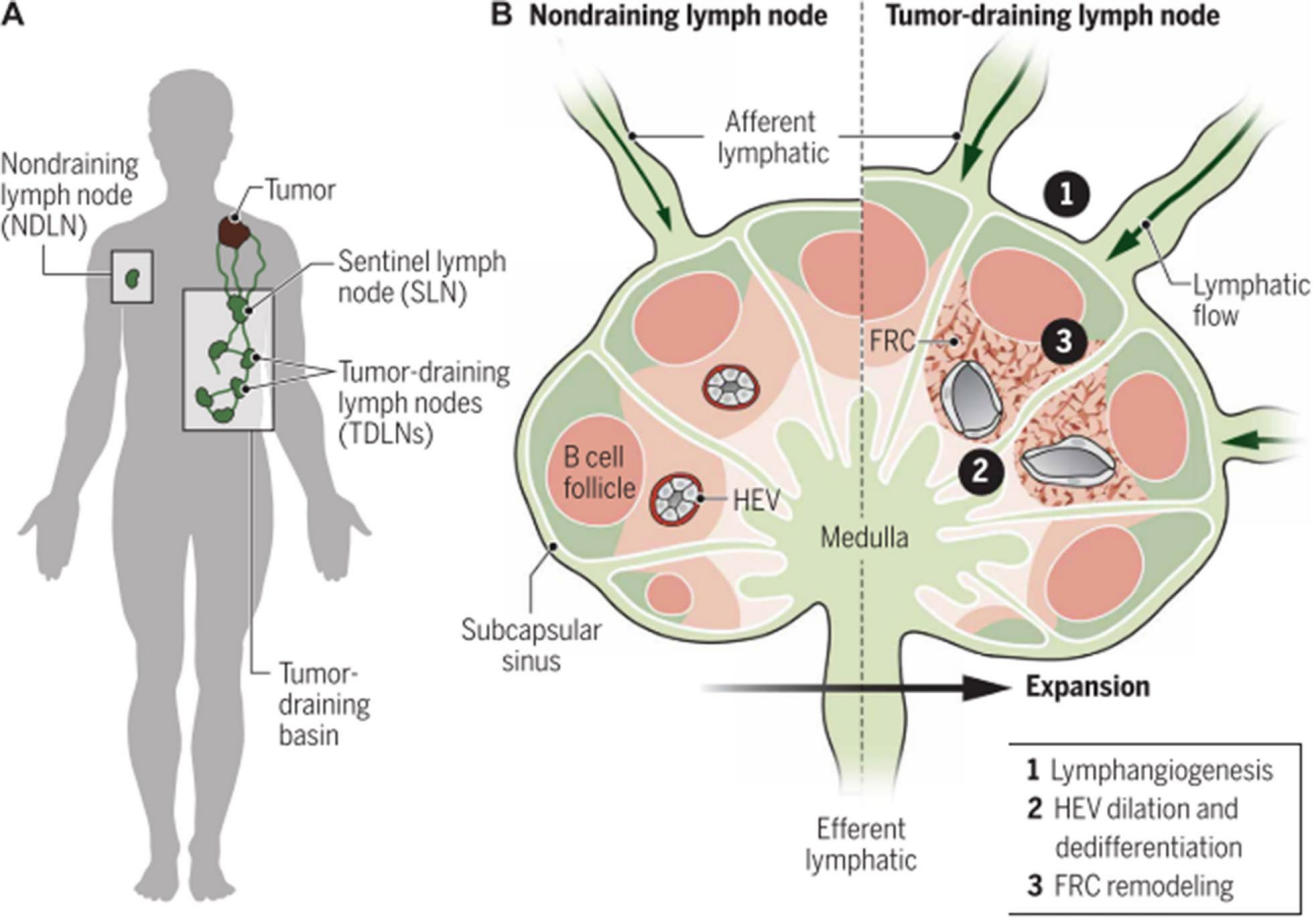
The structural changes of LNs serve as a function of tumor drainage. **A** Solid tumors are connected to LNs through a network of lymphatic vessels that transport fluid, soluble factors, lipids, and cells. **B** Afferent lymphatics flow from the tumor to the TDLN and deliver tumor-derived material, including antigens and extracellular vesicles, to the TDLN. (Adapted with permission from [29]. Copyright © 2021, du Bois, et al. The American Association for the Advancement of Science)

Solid tumors in peripheral nonlymphoid tissues often come into contact with the lymphatic system and functionally connect both regional lymphatic vessels and their draining LNs, leading to metastatic progression (Fig. 3) [29]. Tumors residing in body areas use TDLNs as regional draining LNs, which are the primary sites of the development of anti-tumor immunity. DCs from tumor tissues are exposed to antigens that then migrate through lymphatic vessels to reach TDLNs. These DCs present tumor-associated antigens to T cells, which then enter the bloodstream and reach the site of the primary or metastatic tumor to recognize and kill tumor cells. Thus, TDLNs are key therapeutic targets for immunotherapy and act as reservoirs of immunostimulatory tumor antigens. However, TDLNs exhibit functions that induce metastasis and inhibit immune surveillance by multiple intersecting mechanisms, such as inappropriate activation of intrinsic leukocyte programs and stromal remodeling, which forms an immune suppression environment [30]. During tumor growth, the tumor microenvironment accumulates high concentrations of immunosuppressive cytokines and molecules. Immunosuppressive cells, such as regulatory T cells (Tregs) and myeloid cells, prevail in the tumor microenvironment. Meanwhile, immunosuppressive cytokines and molecules are drained to the TDLNs that diminish anti-tumor responses. Then, TDLNs enter into an exhausted state, which is accompanied by blocked proliferation of cytotoxic T cells (CTLs), increasing regulatory lymphocytes, and anergic status of DCs, allowing tumor cell installation and growth. Activating or revitalizing the immune response within the TDLN can provide important systemic immune protection against tumor recurrence and distant metastasis.

### Tertiary lymphoid structures and their relevance to tumors

**Fig. 4:**
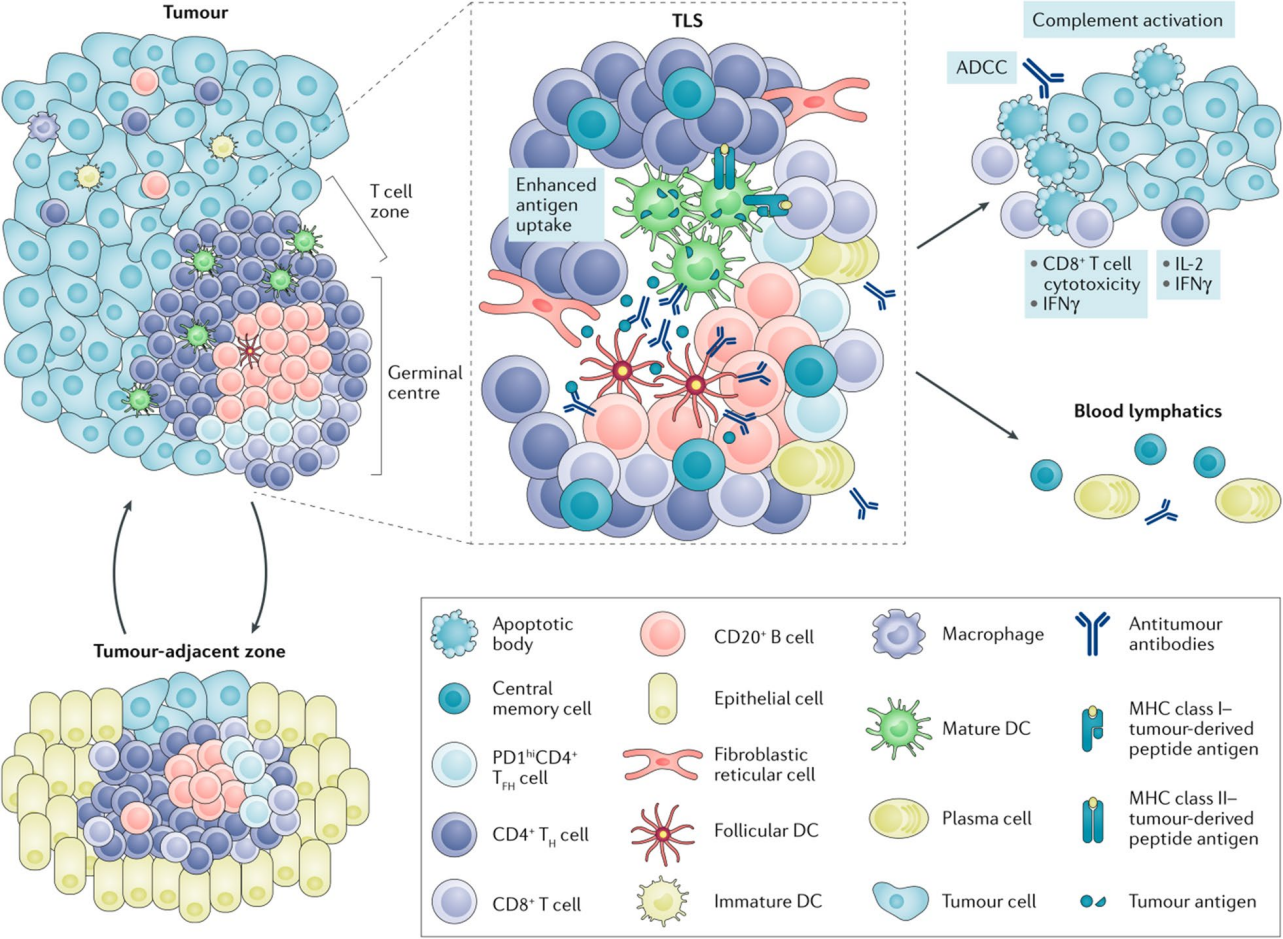
The compositions and functions of tertiary lymphoid structures in tumors. The schematic image shows a tertiary lymphoid structure (TLS) located within a tumor with a CD3^+^ T-cell zone containing DCs, fibroblastic reticular cells (FRCs) and a CD20^+^ B-cell zone. Central memory T and B cells generated in TLSs circulate and avoid tumor metastasis. (Adapted with permission from [31]. Copyright © 2019, Springer Nature Limited)

Tertiary lymphoid structures (TLSs) are the aggregation structures of tissue immune cells located in nonlymphoid tissues and they normally do not occur in normal tissues (Fig. 4) [31]. TLSs can also be considered special LNs inside the tumor due to their same cellular and structural composition as secondary lymph organs (SLOs) [32]. It is commonly referred to as a TLS that is usually formed in tumors, organ transplantation and other chronic inflammatory sites [33, 34]. TLSs have the same APCs and immune response cells as LNs, and more tumor antigens are expressed in TLSs than in TDLNs [35]. The interaction between the tumor and the immune system is often referred to as the “tumor-immunity cycle”, which diagrams the relationship between the tumor and TDLNs in a steady state [36]. When tumor cells die, they first release neoantigens that can be captured by DCs. Then, these DCs migrate to TDLNs to prime and activate effector CD8^+^ T cells against tumor-specific antigens. Finally, these T cells migrate to the tumor sites and kill tumor cells expressing the same antigen through the interaction between the T-cell receptor (TCR) and its cognate antigen bound to MHCI. Dead tumor cells release more neoantigens and form a new cycle [36, 37]. During this cycle, CD103^+^/CD141^+^ DCs bearing CCR7 play a key role in delivering tumor antigens to TDLNs; furthermore, the paucity of CCR7 can lead to blocked T-cell initiation with tumor growth, and the loss of activated CD103^+^ DCs in tumors will reduce the efficacy of checkpoint blockade [38, 39]. Meanwhile, the tumor microenvironment may suppress the above effector cells and thus disrupt the antitumor immune response [40, 41].

TLSs are thought to promote the recruitment and activation of tumor antigen-specific T cells, while tumor-infiltrating T cells may also be the cause of TLS formation through CXCL13-mediated recruitment of immune cells [42]. As a site of memory cells that generate circulating effects to control tumor recurrence, TLSs also play a role in tumor immunotherapy [31]. For example, intratumoral B cells associated with TLSs have been shown to improve the response to ICB therapy and prolong the survival time in melanoma patients [43]. In another study, the clinical response of patients with cervical intraepithelial neoplasia (CIN) was related to the induction of TLSs in the regression of lesions after treatment with human papilloma virus (HPV) vaccination [44]. Therefore, TLSs may not only be used as a prognostic marker for the response to tumor immunotherapy but also promote tumor immune responses. The development of methods for administering chemokines, antibodies or APCs to target TLSs in tumors is also a potential strategy for promoting tumor immunotherapy.

### Interaction between LNs and nanovaccines

The efficient penetration of T cells and antibodies is inhibited by the physiological barriers of tumor tissues [45, 46]. In contrast to adoptive T-cell and ICB therapies, the target of nanovaccines is LNs rather than tumors, so they can reduce the impact of physiological barriers in tumor tissues on the delivery efficacy of nanovaccines [47, 48]. Nanovaccines are expected to produce robust CD8^+^ T-cell immune responses [49, 50]. The passive pathway involves interstitial drainage and antigen “depot” formation by programming the properties (e.g., size, shape or deformability) of nanovaccines to passively target LNs, while the active pathway mainly involves targeted ligand modification of nanoplatforms. In addition to the above transport methods, nanovaccines can also be injected directly into LNs. Although this direct method is more convenient than active and passive transport methods that depend on transport mechanisms, the complexity of intranodal injection, which usually requires surgical intervention remains unresolved, and the size of LNs also limits the amount of injection [51–55]. Thus, the development of nanovaccines capable of inducing durable and effective adaptive immune responses is critical for combating future emerging pathogens and designing the next generation of cancer immunotherapies. As adaptive immune responses are initiated primarily in the LNs, the efficacy of nanovaccines is in turn closely linked to their ability to reach and accumulate in the LNs that drain the site of immunity [56]. By specifically targeting LNs, researchers can develop more effective nanovaccines against cancer. Based on their intrinsic potential to focus therapeutic payloads on relevant immune cells and limit systemic distribution, a series of advanced biomaterials have been intensively explored to improve the efficacy and safety of vaccines and immunotherapies. In the next section, we will describe how to modify the parameters of nanoplatforms for better targeting LNs.

## Parameters of nanoplatforms for lymphatic targeting

Nanoplatforms have been exploited as delivery vehicles to modulate immune responses and improve the treatment of tumors by potentiating vaccination efficacy [57]. These nanoplatforms possess unique properties and advantages, especially targeting functions. Nanoplatforms can significantly lower the side effects induced by immunomodulatory agents by specific targeted delivery to select lymphoid tissues or immune cells. Meanwhile, this targeted delivery can increase the potency of these agents, reducing the required dose to evoke enough immune responses. In addition, nanoplatforms can protect and stabilize agents in vivo or enable co-delivery of antigens and immunomodulatory agents in a single carrier. Various engineering methods have been applied to adjust nanoplatform parameters including surface modification, size, shape and deformability to promote immune responses and reduce the safety concerns of nanovaccines. Representative parameters of nanoplatforms affecting lymphatic targeting are shown in Table 1.

**Table 1:** Representative parameters of nanoplatforms affecting lymphatic targeting

Parameters of nanoplatforms	LN targeting methods	Mechanism of LN targeting	Refs.
Surface targeting ligand	Mannose modification	Targeting of CD206 on DCs in LN	[58]
	Trimannose modification	Targeting of CD206 on DCs in LN	[59]
	Anti-DEC205 modification	Targeting of CD205 on DCs in LN	[60]
Surface charges	Negative or neutral charges	Repulsive interaction with extracellular matrix charges	[61]
	Positive charges	Efficient uptake by APCs	[62]
Surface hydrophobicity	Hydrophilic surfaces	High affinity for interstitial water channels	[63]
Sizes	Adjustment diameters ranging from 10 to 100 nm	Passively entering lymphatic capillaries through endothelial connections	[64, 65]
Shapes	Spherical shapes	More efficient uptake by APCs than rod-shaped and cubic nanoplatforms	[66]
Deformability	Softness	Efficiently crossing the lymphatic endothelium and highly efficient uptake by APCs due to flexible mechanical properties	[67]

### Surface modification of nanoplatforms for LN targeting

#### Surface targeting ligand modification

**Fig. 5:**
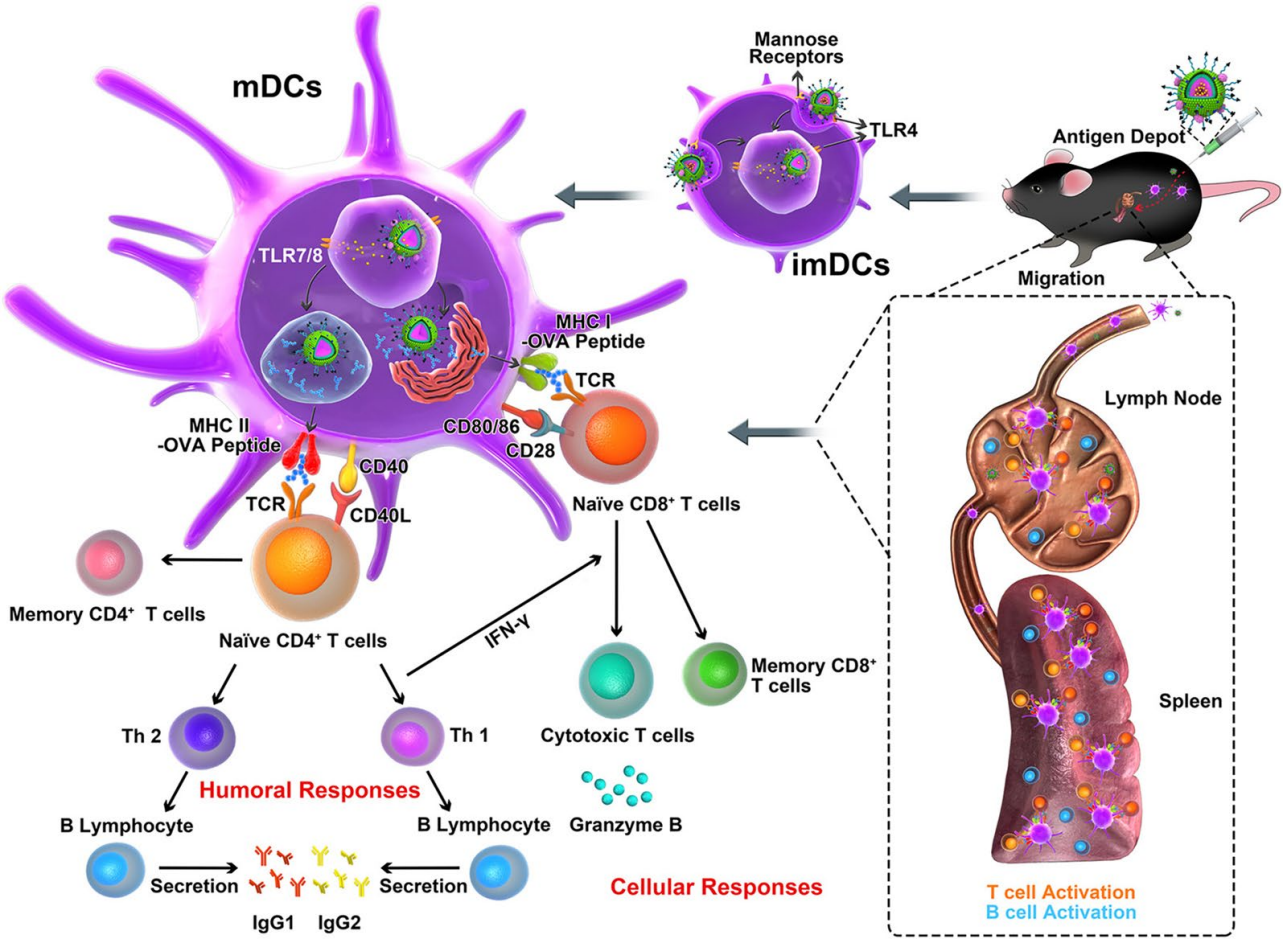
Hybrid nanoparticle-modified targeting ligands promoted trafficking to secondary lymphoid organs. LN targeting and anti-tumor mechanisms of MAN-OVA-IMNPs. (Adapted with permission from [58]. Copyright © 2019, American Chemical Society)

By adding the active targeting ligand on the surface of nanoplatforms, nanovaccines can be constructed for actively targeting LNs. DCs, as the most crucial and potent APCs, play an important role in the initiation of antigen-specific immunity. However, evidence indicates that antigen delivery without targeting and proper stimuli to DCs leads to immune tolerance. Thus, an increasing number of studies have attempted efforts to develop nanovaccines that can actively target DCs in LNs. LN targeting can be achieved by modifying nanoplatforms with antibodies or ligands specific to DCs. It is known that there is high expression of mannose receptor (MR, CD206) and C-type lectin receptor (DC-SIGN, CD209) on the surface of DCs [68–70]. The MR preferably binds mannose and trimannose. Therefore, mannose-modified nanoplatforms are supposed to actively target DCs in LNs. For example, Zhang et al. elaborately designed a novel versatile and mannose-targeting nanovaccine (MAN-OVA-IMNPs) based on the biodegradable polymer PCL-PEG-PCL and cationic lipid DOTAP to increase the effects of tumor immunotherapy (Fig. 5) [58]. They found that MAN-OVA-IMNPs could actively target LNs and be internalized by DCs via mannose decoration to induce antigen-specific CD4^+^ and CD8^+^ T cells to improve both humoral and cellular immune responses. For trimannose-modified nanoplatforms, Kramer et al. demonstrated that trimannose is a more robust targeting unit because its receptor is less easily blocked with simple carbohydrates [59]. In their study, the nanovaccine was composed of SIINFEKL, L18-MDP and p(HPMA)-b-p(LMA)-ran-p(HCMA), with carbohydrates mannose and trimannose introduced into the hydrophilic corona as DC targeting units. Benefiting from the trimannose ligand, the nanovaccine can actively target DCs and promote antigen-specific CD8^+^ T-cell proliferation. There is also a C-type lectin receptor DEC-205 (CD205) that is expressed only on lymphoid DCs, but not on other peripheral blood mononuclear cells [71]. Shen et al. generated a trifunctional nanovaccine based on ferrous NPs that could target and activate CD8^+^ DCs. The ferrous nanoplatforms can be loaded with immunostimulatory CpG-oligonucleotides, anti-DEC205 antibody and antigen OVA to produce a robust immune response. Vaccination of B16/OVA tumor-burdened mice with the nanovaccine showed tumor growth arrest and prolonged the survival rate. This approach of actively targeting DCs in LNs by modifying targeting ligands on the surface of nanoplatforms is an effective strategy for modulating the immune response at the single-cell level, not only to improve the efficiency of vaccine delivery but also to specifically deliver loaded immunomodulatory reagents to a subset of immune cells to reduce non-specific toxicity [60].

#### Surface charges of nanoplatforms

**Fig. 6:**
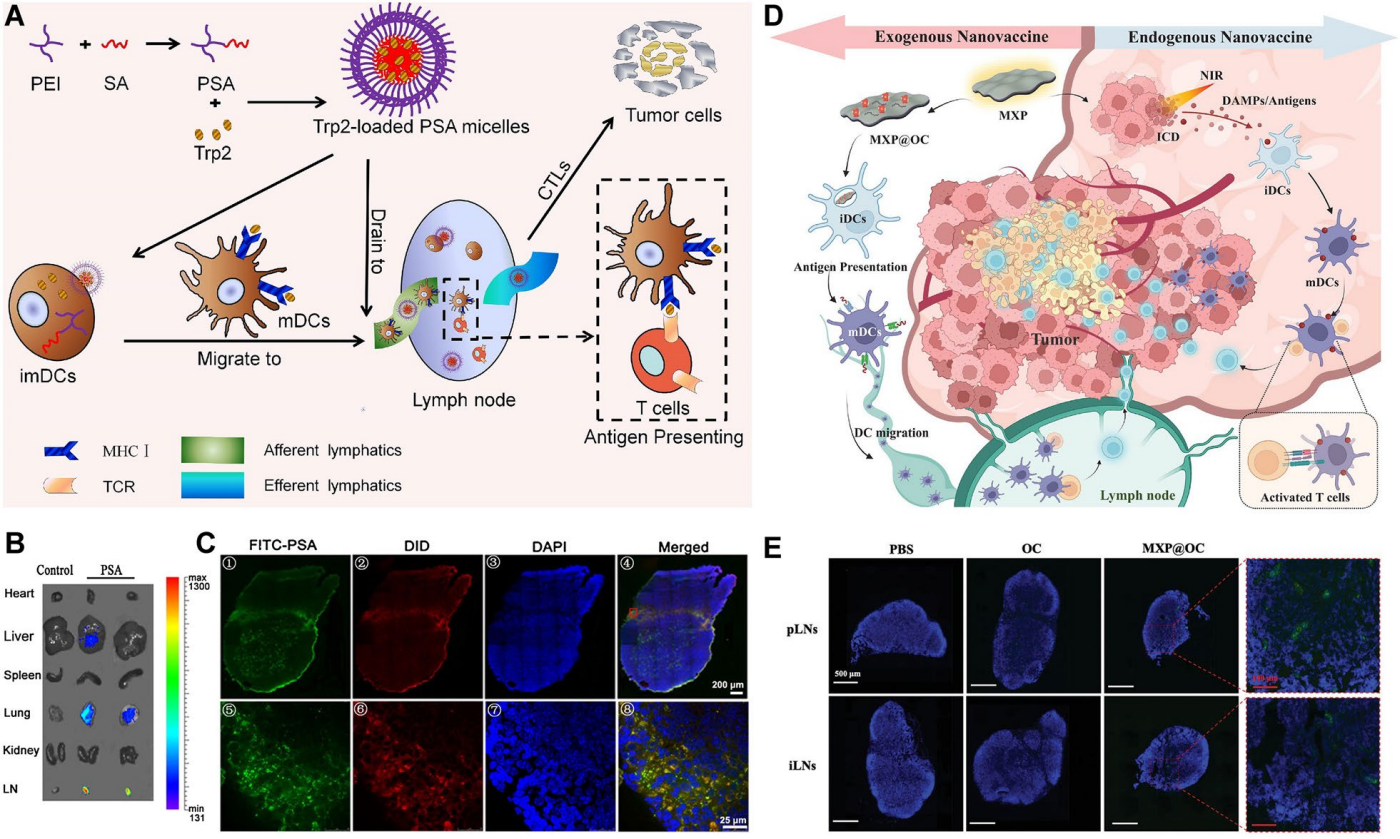
Cationic surface of nanoplatforms for efficient lymphatic draining and enhanced cytotoxic T-lymphocyte responses. **A** Schematic representation of cationic PSA micelles loaded with a Trp2 peptide that were delivered to the draining LNs. The in vivo distribution of PSA showed accumulation in the draining LNs and low systemic spread. **B** Fluorescence imaging of various organs and tissues. **C** Confocal laser scanning microscopy showed the co-distribution of green fluorescence in draining lymph nodes. (Adapted with permission from [62]. Copyright © 2014 Elsevier B.V. All rights reserved.) **D** Schematic illustration of the MXP nanoplatform loaded DCs and promoting LN-targeting and activating the DC-based antitumor immune cascade. **E** The MXP@OC vaccine enhances lymphatic drainage of DCs. (Adapted with permission from [75]. Copyright © 2023. The Zhang et al. Wiley‐VCH GmbH)

The stability of nanovaccines and their interaction with biological systems can be influenced by adjusting the ratio of cationic and anionic nanoplatform-forming materials or by coating the surface of nanoplatforms with materials with different electrical charges, but there is no consensus on which charge is more favorable for LN targeting of nanovaccines. The extracellular matrix (ECM) is mainly composed of a network of collagen fibers, a gel phase of glycosaminoglycans (GAGs), a salt solution and plasma proteins [72]. When nanoplatforms cross the stroma, they are affected not only by the water channel with a diameter of 100 nm in the interstitium that can transport nanoplatforms to the capillaries but also by the charged components in the extracellular matrix. As the main components, GAGs are negatively charged, so nanoplatforms with positive charges will be blocked when they enter the lymphatic capillaries and form depots at the injection site that slowly enter the lymphatic vessel or migrate to LNs with the help of APCs in the stroma. In contrast, nanoplatforms with negative or neutral charges can be transferred more efficiently using hydrophilic biomaterials [73]. In one study, lipid-based nanoparticles (LNPs) with the best size (diameter: 30 nm) were first screened out, and then the LN targeting ability of LNPs with a diameter of 30 nm that carried positive, negative, and neutral charges was evaluated by adding DOTAP (a cationic lipid) or CHEMS (an anionic lipid). The results showed that Neg-LNPs (− 11.9 ± 1.0 mV) have better permeability and higher accumulation levels in LNs than Neu-LNPs (2.4 ± 0.6 mV) and Pos-LNPs (14.5 ± 1.5 mV) [61]. Therefore, they supposed that nanoplatforms with negative charges can better target LNs. However, nanoplatforms with positive charges can be better captured by DCs [74]. Using positively charged nanoplatforms for delivering tumor antigen peptides can reduce systemic dispersion and promote DC maturation and antigen internalization within DCs (Fig. 6A–C) [62]. In addition, some nanoplatforms can promote activated APCs to target LNs for evoking immune responses. Zhang et al. constructed PEI modified Ti_3_C_2_ MXene-based nanoplatforms (MXP) with positively charged surfaces [75]. MXP loaded OVA antigen and CpG (MXP@OC) can facilitate the migration of DCs, which transported into LNs to initiate T-cell activation (Fig. 6D, E).

In addition, the effect of the nanoplatform is also influenced by the type of functional group that controls the surface charge. Nishimoto et al. prepared three anionic dendrimers with carboxyl-, sulfonyl-, and phosphate-terminal groups and then investigated the LN targeting of anionic dendrimers and their association with immune cells in the LNs [76]. The results showed that all three dendrimers with negative charges were present in LNs; among them, generation-5 poly(amidoamine) dendrimers (P-den) with terminal phosphate groups accumulated more strongly in LNs than C-den and S-den dendrimers. P-den can also be recognized by phagocytes and B cells, while the others will flow to the liver. As the most commonly used polymer, PEGylation has a series of advantages: ① improving the bioavailability of nanovaccines; ② avoiding rapid excretion of nanovaccines from the body by preventing nonspecific interactions with blood components (opsonization); and ③ neutralizing the charge of cation and anion carriers to enhance their transport in tissues [77–79]. A dense PEG coating on NPs could cause the surface charge to be neutral to promote transport in the organization [80]. TDLNs are critical sites where T cells are primed for activating immune responses against tumors. Nam et al. developed polyethyleneimine (PEI)-based NP vaccines, which were coated with PEG [81]. They showed that conjugation with PEG reduced the cytotoxicity of PEI and promoted the priming and activation of APCs because colloidal stability was enhanced by the PEG passivation layer. PEGylation of the nanovaccine can reduce tumor retention but enhance strong immune activation in local TDLNs in vivo. Taken together, the surface charges of nanoplatforms play an important role in crossing of the interstitium and in triggering subsequent immune responses.

#### Surface hydrophobicity

As mentioned earlier, nanoplatforms with diameters between 10 and 100 nm can rapidly reach lymphatic capillaries via interstitial water channels after interstitial administration. On this premise, nanoplatforms with hydrophilic surfaces can enter the lymphatic vessels through interstitial drainage to reach LNs more efficiently [63]. In contrast, nanoplatforms with hydrophobic surfaces prefer to form depots the same as positively charged nanoplatforms, which will be intercepted by APCs in the interstitium and then enter into lymphatic vessels together, but this process is relatively slow. Moghimi et al. found that liposomes modified with mPEG2000-lipid could be discharged into the initial lymphatic vessel faster than liposomes modified with mPEG350-lipid after subcutaneous injection in mice due to increased surface hydrophilicity and close association of water molecules with PEG chains. These nanoplatforms interact poorly with the ground substance of the interstitium [82]. In addition, Rao et al. prepared different nanoplatforms with defined sizes and relative hydrophobicity [83]. In their study, the left ileac node (LPN) and total nodal accumulations were determined, and PP nanoparticles (low hydrophobicity) had much higher nodal accumulation than PS nanoparticles (higher hydrophobicity), indicating that hydrophobicity significantly influenced the LN targeting of nanoplatforms.

### Size parameters of nanoplatforms for LN targeting

Optimizing the size of nanoplatforms is considered to be one of the most important methods for controlling the targeted delivery of nanovaccines to LNs [71]. After nanovaccines are administered through interstitial injection, nanoplatforms with diameters less than 10 nm will pass through blood endothelial cells and enter capillaries. Then, these nanoplatforms will be removed from the interstitium because of the high capillary flow velocity. Moreover, although nanoplatforms with a smaller diameter can penetrate LNs more effectively, they have a low correlation with local leukocytes after entering LNs because smaller nanoplatforms are less efficiently absorbed by DCs [84]. Nanoplatforms with a diameter of 10–100 nm can passively enter lymphatic capillaries through endothelial connections because the basement membrane of lymphatic capillaries is discontinuous and lacks smooth muscle, and then these nanoplatforms are transported into LNs through lymphatic drainage and accumulation [64, 65]. A study of NPs based on PPS found that NPs with a diameter of 20 nm could be delivered to LNs more quickly after footpad injection and retained there for 120 h compared with the transport efficiency of NPs with a diameter of 100 nm [85, 86]. Larger nanoplatforms (diameters larger than 100 nm) will be restricted by interstitial water channels with a diameter of 100 nm that connect the blood capillary and lymphatic capillary, so these nanoplatforms mainly stay at the site of drug injection. However, they can still reach the LNs after being ingested by peripheral APCs, although this process is usually slow [87]. In one study, antigens were loaded onto 30 and 500 nm NPs to compare the adaptive immune response. The results showed that although the amount and efficiency of 500 nm NPs reaching LNs were insufficient, they could elicit a stronger CD8^+^ T-cell response [88]. Therefore, the size of nanoplatforms may not only determine how the vaccine enters the LNs but also influence the subsequent immune responses by affecting the distribution of the vaccine in LNs.

### Shape parameters of nanoplatforms for LN targeting

Some scholars have also explored the influence of shape on the transport and uptake process of nanoplatforms. It has been demonstrated that the shape of nanoplatforms can affect the microstructure of the vaccine depot at the injection site if the vaccine cannot enter the LNs in time, thus affecting the LN targeting ability of the vaccine [89]. By adjusting the shape of nanoplatforms, the assembly can be enhanced so that the vaccine depot can recruit more DCs and their uptake of the cargo released by the vaccine can also be improved [89, 90]. For example, Huang et al. developed three kinds of monodisperse mesoporous silica nanoparticles (MSNs) with different aspect ratios (ARs, 1, 2, 4) but the same other components, and their effects on cell uptake and behavior were compared [90]. The results showed that the nanoplatforms with the largest ARs reduced cell viability/apoptosis more severely than other nanoplatforms, which was due to their easy uptake by cell endocytosis. Therefore, spherical inorganic nanoplatforms are more suitable for LN-targeted delivery of vaccines because they remain in the blood circulation for a longer time than short and long rod-shaped nanoplatforms. In addition, for vaccines that directly target LNs without APCs, the shape of nanoplatforms has little effect on vaccine transport but may influence the subsequent immune responses. Hinde et al. compared the ability of four polymer NPs with different shapes but the same surface properties to cross the cell barrier [91]. They found that rod-shaped and worm-shaped nanoplatforms can penetrate the nuclear membrane better than spherical particles, but there was no significant difference in escape through the plasma membrane and endosomes. When the vaccine reaches LNs, the extent of immune responses caused by nanoplatforms with different shapes also varies. For instance, Niikura et al. prepared three kinds of NPs with different shapes (40 × 10 nm spherical NPs, 40 × 10 nm rod-shaped NPs, 40 × 40 × 40 nm cubic NPs) coated with West Nile virus envelope (WNVE) protein to compare their ability to produce antibodies [66]. The results showed that Sphere40 was most effective in inducing antibody production, and both Sphere40 and cubic gold nanoparticles (AuNPs) possessed a low cell uptake rate of APCs but could lead to high secretion of the proinflammatory cytokines TNF-α, IL-6, IL-12 and GM-CSF compared to the rods.

In addition to changing the shape of nanoplatforms to enhance the ability of LN targeting, the antigen loading capacity of DCs in LNs can be further enhanced by expanding the internal space of nanoplatforms [92]. For example, Cha et al. designed extra-large pore mesoporous silica nanoparticles (XL-MSNs) that could accommodate more OVA and Toll-like receptor 9 (TLR9) agonists [92]. After delivering OVA and the TLR9 agonist to LNs by XL-MSNs, the maturation and antigen presentation of DCs were significantly enhanced due to the high loading capacity of large biomolecules of XL-MSNs. In the C57BL/6 mouse model, it was shown that XL-MSNs coloaded with OVA and CpG could produce more antigen-specific CD8^+^ T cells and IFN-γ to induce robust adaptive immune responses compared with the mixture of soluble OVA and CpG, suggesting that XL-MSNs could promote the antitumor effect of nanovaccines through their ability to carry antigens.

### Deformability of nanoplatforms for LN targeting

**Fig. 7:**
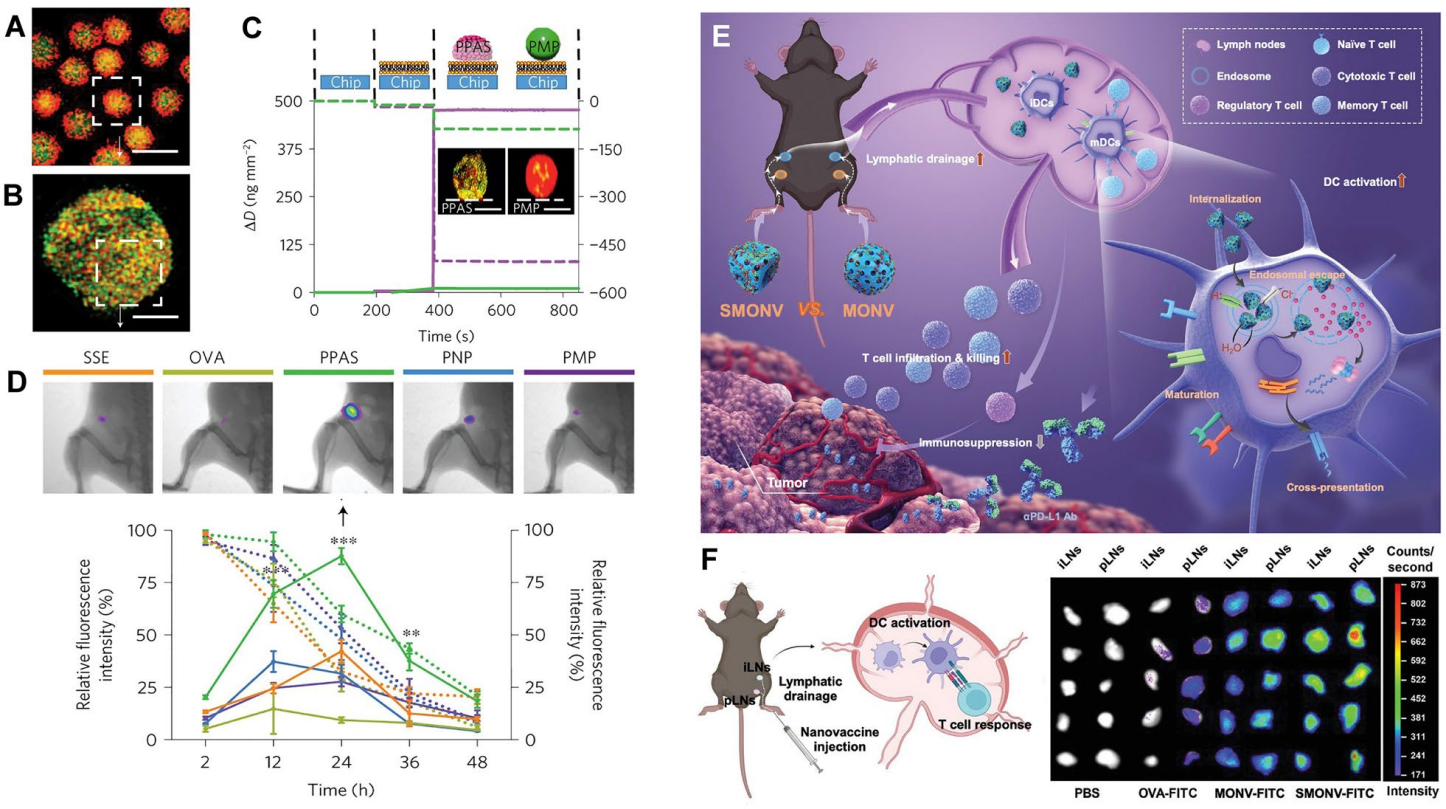
Deformability of nanoplatforms affects LN targeting. **A** Confocal image of PPAS droplets. **B** SIM image of antigen-adsorbed PPAS droplets. **C** QCM-D analysis and corresponding SIM images of the pliability of PPAS and PMPs on the membrane-coated chips. **D** Presence of migrated antigens and quantitative fluorescent intensity of antigens in the draining LNs. (Adapted with permission from [93]. Copyright © 2018, Springer Nature Limited.) **E** Schematic illustration showing that the soft nanovaccine promotes LN targeting and evokes robust DC-mediated antitumor immune responses. **F** Schematic illustration and optical imaging of in vivo lymphatic drainage of the nanovaccines. (Adapted with permission from [67]. Copyright © 2022, Wiley–VCH.)

Nanovaccines with good flexibility can better bind to DCs and promote their transport to LNs. Therefore, improving the softness of NPs can better demonstrate the potential of nanovaccines targeting LNs [8]. For instance, Xia et al. developed Pickering emulsions stabilized by poly-(lactic-co-glycolic acid) (PLGA) NPs and then formed the Pickering Emulsion Adjuvant System (PPAS) (Fig. 7A–C), which retained the force-dependent deformability and lateral mobility of the presented antigens [93]. Benefitting from the pliability, PPAS droplets were able to expand their contact with DCs by settling and deforming on the cell membrane. Although the droplets deformed after cellular wrapping, their integrity was not damaged. After subcutaneous injection, the PPAS formed a strong antigen depot and targeted LNs effectively with the help of DCs. PPAS has also been shown to accumulate in LNs more efficiently than solid NPs (Fig. 7D). Furthermore, Li et al. designed a soft nanovaccine based on mesoporous organosilica (SMONV), and its tumor vaccination cascade was evaluated (Fig. 7E) [67]. Compared with its rigid counterpart MONV, they found that SMONV drained and accumulated more efficiently in LNs after injection into mice (Fig. 7F). Furthermore, they also compared the antitumor effects of SMONV and MONV in a tumor-bearing mouse model. They found that tumor growth was only moderately inhibited in MONV treated tumor-bearing mice but significantly reduced in the SMONV-treated group. The results demonstrated that SMONV was able to deliver antigen to the draining LNs and enhance DC-mediated T-cell immune responses by maximizing antigen exposure in the lymphatic system.

In addition to targeting LNs with DCs, soft nanovaccines can also utilize their deformability to target LNs more effectively across the lymphatic endothelium. For instance, Song et al. developed a deformable vaccine delivery system (DASE) based on an albumin-stabilized emulsion, which was expected to achieve dual LNs-targeting strategies by both intracellular and intercellular routes [94]. After intramuscular administration, some droplets (≈ 330 nm) remained at the injection site to form depots and activate the immune-potentiate microenvironment, and then entered LNs with the help of DCs, while others could automatically adjust their size to pass through the endothelial clefts (20–100 nm) due to the self-adaptive deformability of droplets. Therefore, DASE could target LNs from the injection site more efficiently than solid albumin particles (SAPs), although both of them formed depots. In addition, they also found that DASE induced more DCs, of which 10.6% were resident DCs and 9.37% were recruited from peripheral tissues, indicating that adjusting the deformability of nanovaccines may provide an efficient strategy for effective LN targeting and booster vaccination.

## Advanced nanovaccine delivery systems for LN targeting

The continuous development of advanced delivery systems has improved the efficiency of vaccines targeting LNs. As discussed previously, the delivery of vaccines from the injection site to LNs can be optimized by adjusting the physical or chemical properties of nanoplatforms. Recent developments in LN-targeted nanoplatforms based on different material components are discussed elaborately in the next.

### Nanoplatforms based on liposomes

Liposomes are phospholipid bubbles with a double membrane structure, usually consisting of one or more lipid bilayers, with water phases in and between the bilayers [95]. Liposomes have attracted much attention as vaccine carriers due to their special characteristics: (I) Liposomes have better biocompatibility. (II) Liposomes are easy to modify in size and surface area to better target LNs. (III) LNP-based nanovaccines can protect antigens from degradation in vivo without undesirable side effects. Liposomes usually remain at the injection site due to their positive charge [6, 95]. By increasing the amount of PEG on the surface of positively charged liposomes, the clearance rate of the injection site can be improved and the antigen depot can be reduced so that the vaccine can target LNs more effectively [96]. Representative liposome-based nanovaccines for targeting LNs in tumor immunotherapy are shown in Table 2. For example, although cationic liposomes have been shown to be a new adjuvant and vaccine delivery system, whether enhanced LN targeting will improve the efficiency of cationic liposomal formulations of vaccines has not been elucidated. To investigate the effect of PEGylation on the LN targeting ability and immunogenicity of liposome-based nanoplatforms (LNPs), Zhuang et al. added 1 or 5 mol% 1,2-distearoyl-*sn*-glycero-3-phosphoethanolamine-*N*-[methoxy(polyethylene glycol)-2000] (DSPE-PEG2000) to 1,2-dioleoyl-3-trimethylammoninum propane (DOPTA) cationic liposomes [97]. They finally found that 1% DSPE-PEG2000 in DOPTA cationic liposomes was more effective for LN targeting and activating the rapid and effective antigen-immune responses of nanovaccines compared to 5% DSPE-PEG2000, which prolonged the residence time of DOPTA liposomes in the blood circulation and nonPEGylated DOTAP liposomes. The targeting of nanovaccines carrying tumor antigens and adjuvants to LNs is an attractive approach to improve the outcome of cancer immunotherapy. However, the application of this technology is limited by the lack of suitable tumor-associated antigens (TAAs) and the complex technology required to identify tumor neoantigens [98]. Yu et al. designed a scaffold based on cholesteryl oleate (CO) and 1,2-dimyristoyl-*sn*-glycero-3-phosphocholine (DMPC) and then loaded the scaffold with melittin to form α-melittin NPs and demonstrated that better LN targeting and a robust immune response can be achieved by vaccines that are not loaded with additional tumor antigens [98]. Their results showed that α-melittin NPs could effectively efflux into LNs and activate macrophages and DCs, while the toxicity of melittin to these DCs was reduced due to the good binding ability of DMPC to the cell membrane. Regarding antitumor effects, α-melittin NPs activated tumor-specific T-cell responses and inhibited the growth of primary and distant tumors through cellular and humoral immunity in a bilateral tumor-bearing mouse model. Kuai et al. also designed a high-density lipoprotein-mimicking nanodisc vaccine (sHDL-Ag/CpG) and demonstrated that HDL-mimicking nanodiscs coupled to antigenic peptides and adjuvants significantly improve antigen/adjuvant co-delivery to lymphoid organs and maintain antigen presentation ability of DCs [99]. After subcutaneous injection into the tail root of mice, sHDL-Ag showed an obvious increase in FITC signaling in draining lymph nodes (dLNs), with Ag (FITC) and Cy5-labeled 22A co-localized in dLNs. Meanwhile, Cy5-tagged Cho-CpG in sHDL promoted its accumulation in dLNs more than free soluble injection. These results demonstrated that antigen and Cho-CpG can be delivered together into LNs by sHDL to trigger prolonged Ag presentation and cross-priming of T cells, and then robust and long-lived antigen-specific CD8α^+^ CTL responses were activated with regression of the tumor.

**Table 2:** Representative liposome-based nanovaccines for targeting LNs in tumor immunotherapy

Class of nanovaccines	Size of nanovaccines	Zeta potential of nanovaccines	Active components	Mechanism of targeting LNs	Anti-tumor effects	Refs.
DOPTA-1% PEG	245.83 ± 26.30–267.87 ± 32.68 nm	Positive charge: 15.54 ± 2.41 mV	OVA, DOPTA	Partially shielded surface charge	Enhanced primary and secondary anti-OVA antibody responses	[97]
α-melittin NPs	10–20 nm	Neutral charge	Melittin, CO, DMPC	Efficiently shielded the positive charge of melittin; Optimal size for LN targeting	Activated tumor antigen-specific cellular and humoral immune response; Eliminated both primary and distant tumors in mice	[98]
sHDL-Ag/CpG	10.5 ± 0.5 nm	–	DMPC, Cho-CpG, antigen peptide Ag	Optimal size for LN targeting; Suitable shape for LN targeting	Prolonged Ag presentation on APCs; Inhibited tumor growth by generating broad-spectrum antitumor T-cell responses	[99]
cKK-E12	80–110 nm	Negative charge: − 15 to − 3 mV	Tumor RNA, LPS	Promoting cellular uptake and endosomal escape; Reducing nonspecific interactions in vivo	Produced strong CD8^+^ T-cell responses; Shrank B16F10 melanoma tumors and extended the overall survival of mice	[100]
ssPalm-LNPs	140–180 nm	Negative charge: − 6.4 ± 4.6 mV	pDNA, OVA, ssPalm	Promoting the uptake of DCs	Elicited a strong cytotoxic T lymphocyte activity; Inhibited tumor growth and prolonged the survival time of mice	[102]

In addition, LNPs can also be used for the delivery of nucleic acid vaccines due to their good biodegradability, and their stimuli-responsive characteristics contribute to the efficient delivery of these vaccines into LNs. For instance, given that the intracellular delivery of mRNA vaccines to the cytoplasm of antigen-presenting immune cells is still not well understood, Oberli et al. reported a LNP formulation based on an ionizable lipid for the delivery of mRNA vaccines [100]. Ionizable lipids are neutral under physiological pH conditions but positive under low pH conditions, which can not only bind to negatively charged mRNA, but also promote endosomal escape of LNPs by interacting with the negatively charged lysosomal membrane [101]. By observing the distribution of LNPs in mice through firefly luciferase (FFL) mRNA, it was found to be expressed in draining, inguinal, and some axillary LNs, but not in the liver, spleen, lung, or intestine, indicating that the LNPs were well targeted to LNs. Additionally, the mRNA LNP formulation induced effective tumor immunity, activated specific CD8^+^ T cells and prolonged the survival period of tumor-bearing mice. Furthermore, Maeta et al. also designed LNPs based on a SS-cleavable and pH-activated lipid-like material (ssPalm) to deliver DNA nanovaccines [102]. After the stimulation of GSH in the cytoplasm, the pH/reduction dual-responsive ionizable lipid will rapidly decompose to improve the efficiency of drug delivery [103]. Therefore, the nucleic acid-based nanovaccines could be effectively absorbed by APCs and delivered to LNs, and then the pDNA encoding OVA will be released to activate the CTLs to inhibit the growth of tumors.

### Nanoplatforms based on polymers

Polymeric nanoplatforms containing synthetic or natural polymeric substances have shown great advantages in the field of immunotherapy due to their good biodegradability, structural flexibility, and ease of preparation [104]. One of the most commonly used synthetic polymers is polyethyleneimine (PEI), a kind of cationic polymer [105]. PEI can be bound to heparan sulfate proteoglycans on the surface of APCs and internalized through endocytosis. In the process of endosomal acidification, the endosomes will rupture due to the proton sponge effect of PEI, which can improve the efficiency of nanovaccines reaching LNs [105]. However, the high toxicity of PEI due to its high molecular weight limits its application in vaccine delivery [106]. To overcome this shortcoming, PEI surfaces can be modified to improve biological safety, such as PEGylation (as mentioned above) and grafting fluorocarbon chains [107]. In addition, polymeric hybrid micelles (HMs) can also be given the ability to target LNs by tailoring their physicochemical properties [108]. For instance, Zeng et al. combined polyethyleneimine-stearic acid coupling (PSA) with another amphiphilic diblock copolymer, poly-(ethylene glycol) phosphoethanolamine (PEG-PE) to prepare polymeric hybrid micelles (HMs) by self-assembly [108]. Typically, PEGylated modification of the nanoplatform in a chemically covalent manner may damage the nanoplatform and the proteins loaded on the nanoplatform may be inactivated to some extent and increasing the PEG ratio in PEGylated DOTAP lead to greater liver tropism, where the cationic particle may cause systemic toxicity [97]. Therefore, they used self-assembly of cationic micelles prepared from the amphiphilic copolymer PSA as an alternative to PEG. The sizes of hydrophilic and hydrophobic segments in PEG-PE and PSA were similar, but the charge was opposite, which allowed PEG to be physically introduced into cationic micelles without any complex chemical conjugation. The molar ratio of PEG-PE and PSA affects the kinetics of HMs in vivo and they identified 1:1 as the best ratio that enabled HMs to target LNs efficiently. Therefore, they addressed the problem that nanoplatforms with neutral or negative surface charges between 10 and 100 nm were not efficiently taken up by APCs and were not sufficiently retained in LNs by tuning the physicochemical properties of polymeric HMs. Additionally, some polymers can not only target LNs efficiently but also improve the efficacy of tumor immunotherapy by embedding sensitive bonds [109]. For example, although cancer vaccines against patient-specific neoantigens have emerged as a promising strategy, neoantigen peptides are poorly immunogenic and ineffective in stimulating CD8^+^ T-cell responses. To promote intracellular delivery of neoantigen peptides and CDN STING agonists to enhance CD8^+^ T-cell responses, Shae et al. designed a synthetic tumor nanovaccine platform (nanoSTING-vax) [109]. Among them, tertiary amino groups will be cleaved in a low pH environment after entering cells to promote cargo release, while loading cGAMP into PEG-DBP has no impact on the size or neutral zeta potential of particles. PEG-DBP also has a proton sponge effect that could help nanoSTING-vax escape from endosomes to reach LNs efficiently, and then, the breakage of sensitive bonds further promotes the release of antigen and cGAMP, allowing cGAMP to come into contact with STING. Subsequently, the STING pathway is activated to promote DCs to present and process antigens. Finally, antigen-specific CD8^+^ T cells are primed to destroy tumor cells effectively. Nevertheless, the stimulation of systemic cytokine responses needs to address important questions regarding toxicity and safety. Additional studies are needed to optimize nanoSTING-vax dosing, to modulate the range of systemic distribution and to further understand and manage potential toxicity. In addition to tertiary amino groups, some sensitive bonds can be broken under the stimulation of GSH. Lv et al. decorated redox-responsive hyperbranched poly (amido amine) with polyethyleneimine (600 Da) (PEI600) to form PAA-PEI600 and then used it together with partially carbonized PAA-PEI600 PDs to load OVA antigen as tumor nanovaccines (PDs/OVA) [110]. The redox-responsive-S–S-in the vaccine carriers could utilize the concentration difference of glutathione (GSH) inside and outside the cells and the PDs possess good biocompatibility and low toxicity. After the vaccine enters cells, the high concentration of intracellular GSH will cause the disulfide bond to breakdown and promote the release of cargo. In conjunction with the proton sponge effect of polycations, PDs/OVA can efficiently target LNs and release antigens to promote the maturation of DCs to trigger immune responses; at the same time, it can also increase the production of IL-12 and INF-γ to enhance cellular immunity. Furthermore, the tumor-bearing mouse model inoculated with E.G7-OVA cells showed that PDs/OVA can produce significant therapeutic effects.

Furthermore, some polymers can also be modified by active targeting ligands to construct nanovaccines. Whole tumor cell lysates (TCLs) have been implemented as tumor antigens for cancer vaccine development, but the clinical results of TCL-based antitumour immunotherapies remain unsatisfactory. Due to the high expression of mannose receptor (MR, CD206) on the surface of DCs [68], Shi et al. modified the natural polymer chitosan with mannose and then loaded TCLs from B16 melanoma cells as antigens to form a nanovaccine (Man-CTS-TCL NPs) that specifically targeted DCs [111]. Compared to unmodified mannose vaccines (CTS-TCL NPs and TCL alone), Man-CTS-TCL NPs can better promote DC maturation and target LNs more effectively to produce strong cellular and humoral immunity through the targeting effect of mannose. In the mouse model bearing B16 tumors, vaccination with Man-CTS-TCL NPs reduced the tumor mass and increased the number of CD8^+^ T cells in the spleen, indicating that Man-CTS-TCL NPs possessed great antitumor effects. Despite the remarkable characteristics of chitosan as a vaccine delivery platform, one of its main limitations in biomedical applications is its low solubility under physiological conditions [112], and future research will focus on addressing its low water solubility, irregular particle size distribution and low target specificity under physiological conditions. Representative polymer-based nanovaccines for targeting LNs in tumor immunotherapy are shown in Table 3.

**Table 3:** Representative polymer-based nanovaccines for targeting LNs in tumor immunotherapy

Class of nanovaccines	Size of nanovaccines	Zeta potential of nanovaccines	Active components	Mechanism of targeting LNs	Anti-tumor effects	Refs.
HMs	22.6 ± 1.15 nm	Negative charge: 31.40 ± 1.37 mV	Trp2, CpG OND	Tailoring the physicochemical composition	Expanded antigen specific cytotoxic T lymphocytes; Inhibited tumor growth in lung metastatic melanoma model	[108]
nanoSTING-vax	20–100 nm	Neutral charge	cGAMP, OVA-derived peptide SGLEQLESIINFEKL, PEG-DBP	Proton sponge effect; Embedding of sensitive bonds	Complete tumor rejection; Durable antitumor immune memory	[109]
PDs/OVA	70.27 ± 0.51 nm	Positive charge: 37.98 ± 0.60 mV	PAA-PEI600, OVA	Proton sponge effect; Embedding of sensitive bonds	Inhibited tumor growth of the mice bearing E.G7-OVA tumor and prolonged their survival time	[110]
Man-CTS-TCL NPs	120 nm	Negative charge: − 12 mV	Mannose, tumor cell lysates	Targeted ligand modification	Enhanced cytotoxic T lymphocytes responses against tumor; Significantly delayed tumor growth in mice	[111]

### Nanoplatforms based on inorganic substances

In recent years, a series of inorganic nanomaterials that have inherently exceptional physicochemical properties, including gold-based nanoplatforms, iron-based nanoplatforms, silica-based nanoplatforms and carbon-based nanoplatforms, have been exploited for the delivery of nanovaccines due to the ease of modification and preparation and their special properties such as optical, thermal, electrical and magnetic characteristics [113, 114]. Representative inorganic substance-based nanovaccines for targeting LNs in tumor immunotherapy are shown in Table 4.

**Table 4:** Representative inorganic nanoplatforms for targeting LNs

Class of nanovaccines	Size of nanovaccines	Zeta potential of nanovaccines	Active components	Mechanism of targeting LNs	Anti-tumor effects	Refs.
AuNP-OVA	84.3 ± 0.9 nm	–	Au, OVA	Suitable size for the delivery to LNs	Promoted significant antigen-specific responses; Inhibited tumor growth and prolonged survival in both prophylactic and therapeutic in vivo tumor models	[115]
HA-OVA AuNPs	175.57 nm	Negative charge: − 27.23 mV	HA, OVA, Au	Targeted ligand modification; Light activation enhanced delivery	Promoted MHC I antigen presentation and the cytotoxic T lymphocytes response; Inhibited tumor growth under laser irradiation in mice	[116]
MINPs	261.1 nm	Positive charge: 5.66 mV	SPIO, CpG ODN	Imaging guidance; Promoting the maturation of DCs	Effective photothermal destruction of the primary tumors Reduced both the remaining and distant metastatic tumors in mice	[117]
MWNTs	112 ± 82 nm	Negative charge: − 41.9 mV	CpG, anti-CD40 Ig, OVA	Enhancing the uptake of DCs	Significantly reduced tumor size and lung metastasis in mice	[118]
MSN-R848-OVAp	280 nm	Negative charge: − 50 mV to + 20 mV	Toll-like receptor 7 and 8 agonist R848, OVA	Promoting the maturation of DCs	Generated antigen-specific T-cell response; Improved the pharmacokinetic profile of R848 in mice	[119]

#### Gold-based nanoplatforms

Among inorganic materials, gold-based nanoplatforms have shown great prospects in the field of vaccine delivery. They can adjust their size or surface modification to optimize delivery to LNs, be functionalized with related molecules for regulating immune responses and have been shown to act as adjuvants in vaccination [115]. For example, the potential of gold nanoparticles (AuNPs) has yet to be assessed in the in vivo application of peptide cancer vaccines, so Almeida et al. hypothesized that the immune distribution and adjuvant quality of AuNPs could be used to facilitate the delivery of OVA peptide antigen and CpG adjuvant and enhance their therapeutic efficacy in a B16-OVA tumor model and loaded OVA peptide and CpG1826 onto 30 nm AuNPs that were coated with PEG, and then formed AuNP-OVA and AuNP-CpG (diameter less than 100 nm) to evaluate their therapeutic effect in the B16-OVA tumor model [115]. PEGylation enabled AuNPs to reach LNs safely and promoted their transport in tissues. Meanwhile, it was found that AuNP-OVA treatment exhibited a robust antitumor effect to inhibit tumor growth in mice without adjuvants, implying that AuNPs could act as adjuvants to induce antitumor immune responses when antigens are loaded alone, while avoiding the potential toxicity of high-dose adjuvant. Additionally, some scholars have also made use of the photothermal effect of AuNPs to prepare nanovaccines. Cao et al. decorated AuNPs with hyaluronic acid (HA) and loaded them with OVA antigen to form the HA-OVA AuNP vaccine (175.57 nm, − 27.23 mV) [116]. HA, similar to mannose, could help HA-OVA-AuNPs actively target DCs in LNs by binding to their surface receptor HA to target LNs more efficiently. Moreover, Au NPs under laser irradiation could lead to the destruction of the endosomal membrane and efficient activation of BMDCs. In the EG.7-OVA tumor-bearing mouse model, HA-OVA-AuNPs significantly promoted MHC I antigen presentation and the CTL response to suppress tumor growth.

#### Iron-based nanoplatforms

**Fig. 8:**
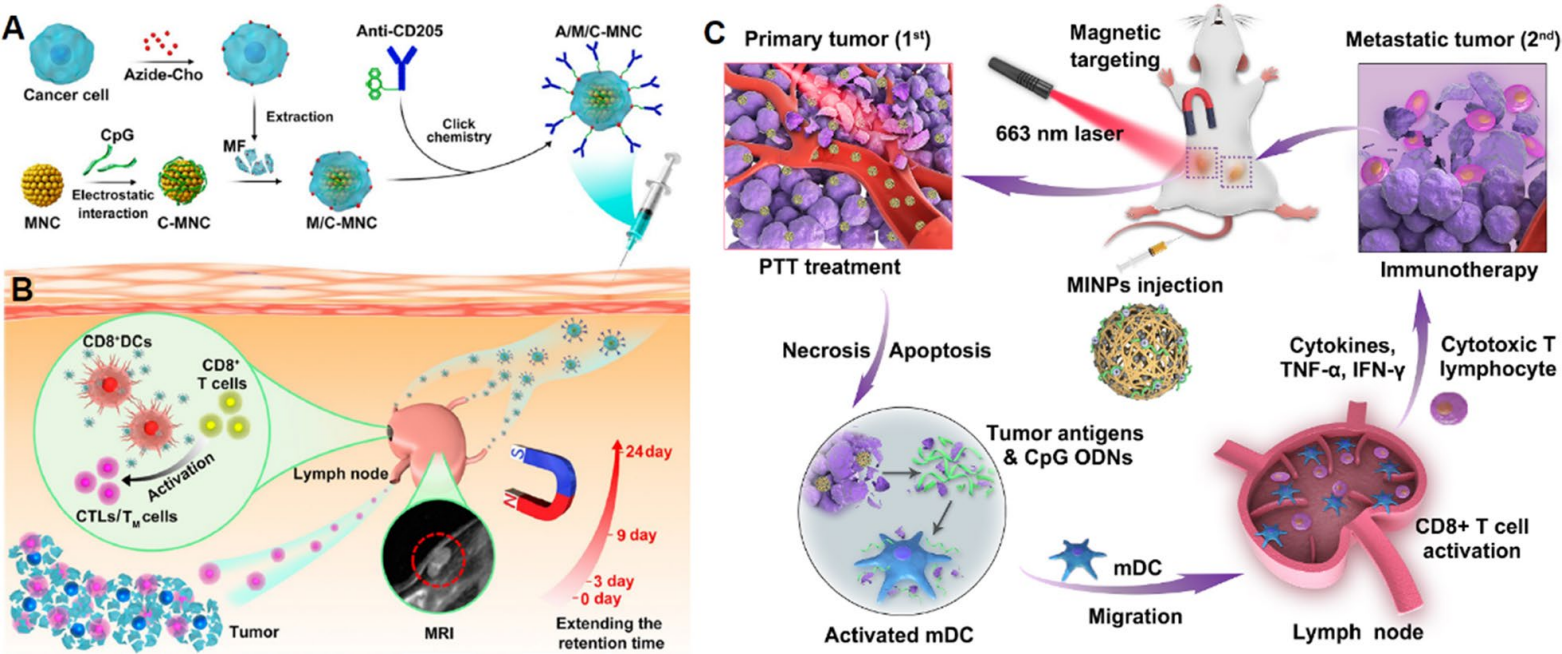
Iron-based nanoplatforms promoting LN-targeting. **A** Fabrication process of A/M/C-MNC. **B** Illustration of A/M/C-MNC-mediated cellular immune responses eliciting cytotoxic T lymphocytes (CTLs) and memory T cells (TM cells) for cancer immunotherapy. (Adapted with permission from [121]. Copyright © 2019 American Chemical Society). **C** Schematic illustration of imaging-guided photothermally triggered immunotherapy based on magnetic-responsive immunostimulatory nanoagents. (Adapted with permission from [117]. Copyright © 2019 Elsevier Ltd. All rights reserved.)

Another special material is ferro-based magnetic nanoplatforms containing superparamagnetic or ferromagnetic Fe_3_O_4_ and Fe_2_O_3_ nanoparticles, pure iron nanoparticles and CoFe_2_O_4_@MnFe_2_O_4_ nanoparticles [120]. Compared with other inorganic materials, ferro-based NPs have the properties of magnetic resonance imaging and can also be used for magnetic hyperthermia and magnetic navigation [120]. Taking advantage of these features, Li et al. developed a novel tumor vaccine based on Fe_3_O_4_ magnetic nanoclusters (MNCs) for safe and powerful tumor vaccination [121]. Fe_3_O_4_ magnetic nanoclusters (MNCs) enabled the vaccine to be held in LNs with MRI guidance (Fig. 8B). Using the click chemistry method, they formed tumor-cell-membrane-coated CpG-ODN-loaded MNCs with anti-CD205 decoration (A/M/C-MNC) (Fig. 8A). They found that A/M/C-MNC targeted LNs efficiently in mice and that the decorated anti-CD205 directed more vaccines to be recognized by CD8^+^ DCs, which further activated CTLs and invoked robust antitumor effects. Furthermore, five different tumor models demonstrated the potent prophylactic and therapeutic effects of A/M/C-MNC, and lung and spontaneous tibia metastasis in 4T1-tumor bearing mice were also inhibited after A/M/C-MNC vaccination. In addition, while theranostic nanoparticle (TNP)-based photothermal therapy (PTT) exhibits prominent promise for cancer therapy, metastatic cancers remain one of the main obstacles to effective PTT. To address this issue, Guo et al. developed magnetic-responsive immunostimulatory nanoagents (MINPs) based on superparamagnetic iron oxide (SPIO) NPs and combined them with cytosine-phosphate-guanine oligodeoxynucleotides (CpG ODNs) [117]. Benefitting from CpG ODNs, the photothermal effects of MINPs could enhance the maturation of DCs and promote their migration to LNs. During this process, SPIO can not only support MRI and PA imaging but also enhance the efficacy of photothermal therapy (Fig. 8C). Through magnetic targeting, MINPs could precisely target TDLNs. After near infrared laser irradiation, the photothermal effect produced by SPIO promoted the release of CpG ODNs, which could reduce the leakage of CpG ODNs elsewhere in the body due to hyperthermia-driven release. Subsequently, CpG ODNs can generate robust T-cell responses with the help of mature DCs and promote the release of proinflammatory cytokines. The toxicity of MINPs was also evaluated in the Balb/c mouse model, and the results showed that MINPs possessed desirable biocompatibility. Liu et al. also demonstrated that SPIO was biocompatible and SPIO-loaded DCs promoted the migration of DCs into LNs [122]. In their study, SPIO-labeled and unlabeled EGFP-DCs were injected into the footpads of mice to evaluate the impact of SPIO on the migration of DCs. The fluorescence imaging results showed that stronger green fluorescence from EGFP-DCs appeared in the inguinal lymph nodes (ILNs) on days 4 and 7 in the SPIO-labeled group than that in the SPIO-unlabeled group, indicating that SPIO could facilitate EGFP-DCs to reach the secondary LNs.

#### Silicon-based nanoplatforms

Silica-based nanoplatforms are another excellent carrier of nanovaccines due to their adjustable pore size, which can improve the drug loading ability [123–125]. To understand which pore size of silica nanoparticles is most suitable for the delivery of vaccines, Hong et al. developed three kinds of mesoporous silica nanoparticles (MSNs) with pore sizes of 7.8 nm, 10.3 nm, and 12.9 nm and loaded OVA antigen onto them to generate OVA@MSNs-S, OVA@MSNs-M and OVA@MSNs-L [126]. After subcutaneous administration, they found that all of them could drain to LNs, and there was no significant difference in the process of uptake of the three Cy5-OVA@MSNs by DCs after they drained into LNs. However, in the subsequent immune responses, OVA@MSNs-L showed a better treatment result than the others due to their better ability to cross-present antigen, which caused them to produce more IFN-γ, IL-4 and TNF-α secreted by CD8^+^ and CD4^+^ T cells. In addition, the results also showed that MSNs with large pore sizes decomposed faster after reaching LNs to promote cargo release, which may be another reason why they can induce a stronger antitumor immune response [126]. Furthermore, MSNs with a large surface area, tunable particle and pore size, and spatially controlled functionalization have been proven to be a safe and versatile carrier system. To investigate whether MSNs can target the delivery of immunomodulators to LNs, Wanger et al. utilized pH-responsive groups to modify SiNPs to promote lysosomal escape of nanovaccines [119]. They designed spatially segregated core–shell MSNs as a pH-responsive drug carrier system for the antitumor immune-stimulant R848 (resiquimod) which is a synthetic Toll-like receptor (TLR) 7 and 8 agonist, and then developed a nanovaccine MSN-R848-OVAp. After subcutaneous administration into mice, the particles accumulated in migratory DCs in the draining LNs and strongly enhanced the activation of the DCs.

#### Carbon-based nanoplatforms

Among carbon-based nanoplatforms, carbon nanotubes (CNTs) and graphene oxides (GOs) are the two most widely used vaccine carriers due to their surface absorption capacity, photothermal effect and immunoregulation capability [120, 127]. For example, multiwalled carbon nanotubes (MWNTs) have shown outstanding potential as tumor antigen nanocarriers; however, the application of MWNTs in the co-delivery of antigens with different types of immune adjuvants to APCs has not been investigated. With this in mind, Hassan et al. loaded CpG and anti-CD40 Ig (αCD40) as adjuvants and OVA as an antigen onto nanotubes and found that MWNTs conjugated with the model antigen OVA could promote their cellular internalization into APCs, which could enhance their delivery to LNs [118]. After subcutaneous injection of the vaccine to evaluate its therapeutic effect in the bearing B16F10 mouse model, CD8^+^ T-cell responses and anti-OVA antibodies were produced in mice, which resulted in a significant reduction in tumor size and lung metastases, indicating that MWNTs were able to remarkably improve the ability of co-loaded OVA, CpG and αCD40 to inhibit the growth of melanoma cells. The stimulation of T cells with potent antitumor properties requires a complex multi-step process that is difficult to achieve with conventional vaccination methods. Xu et al. utilized functionalized-GO to prepare the LN-targeting vaccine RGO(CpG)-PEG-Adpgk, which allows direct delivery of neoantigens and adjuvants to lymph nodes (LNs) and efficient induction of neoantigen-specific T-cell responses [128]. In their study, GO was modified with PEG to form a PEGylated reduced graphene oxide nanosheet (RGO-PEG) that is a highly modular and biodegradable platform. The antigen Adpgk and RGO-PEG were coupled by a Michael addition reaction and then absorbed with CpG to form RGO(CpG)-PEG-Adpgk. Compared with soluble vaccines, RGO(CpG)-PEG-Adpgk exhibited more rapid, efficient, and sustained accumulation in LNs (> 100-fold). Furthermore, RGO-PEG can also induce intracellular ROS in DCs to assist in antigen processing to T cells. In the B16F10 melanoma model, the robust T-cell responses primed by RGO(CpG)-PEG-Adpgk may act synergistically with anti-PD-1 ICB therapy to effectively inhibit the growth of B16F10 tumors.

### Naturally derived nanoplatforms

The usage of naturally derived nanoplatforms intended for interface with immune systems has continued to increase. These naturally derived nanoplatforms contain biological components or cell-derived vesicles, which have high loading capacity as immunomodulators, desired specific cellular uptake, and low toxicity. Representative naturally-derived nanoplatforms for targeting LNs in tumor immunotherapy are illustrated in Table 5.

**Table 5:** Representative naturally derived nanoplatforms for targeting LNs

Class of nanovaccines	Size of nanovaccines	Zeta potential of nanovaccines	Active components	Mechanism of targeting LNs	Anti-tumor effects	Refs.
Man-RBC-NPs	156.6 ± 4.6 nm	Negative charge: − 20.1 ± 0.7 mV	Mannose, MPLA, hgp10025-33	Targeted ligand modification	Prolonged tumor-occurring time; Suppressed tumor growth and metastasis in mice	[129]
DCM/HCtSA/OVA	105.41 ± 2.61 nm	Negative charge: − 5.63 ± 0.31 mV	DCM, OVA	Homologous targeting DCs in LNs	Induced potent T-cell immune responses; Promoted secretion of antitumor-related cytokines	[130]
MOF@FM	145.6 ± 10 nm	Negative charge	FMs	LN-homing capacity	Generated powerful antitumor immune response by direct and indirect T-cell activation in mice	[131]
CpG-SAV-exo	109 ± 10 nm	Negative charge: − 32 ± 1.6 mV	CpG, SAV	Promoting the uptake of APCs	Exhibited stronger in vivo antitumor effects and inhibited tumor growth in mice	[132]
EXO-OVA-mAb	95.6 nm	-	mAb, immunostimulatory EXO	CTLA-4 functionalization of EXO; Optimal size for LN targeting	Increased the ratio of cytotoxic T lymphocytes (CTLs)/Treg	[133]
PVP-MPDA@R837	190.1 nm	Negative charge: − 3.54 ± 0.27 mV	PVP, R848	Using R837 as the model immunomodulatory; Reducing nonspecific interactions in vivo	Induced robust immune activation against tumor cells with photothermal effects of PDA in mice	[134]

#### Nanoplatforms camouflaged with immune cell membranes

**Fig. 9:**
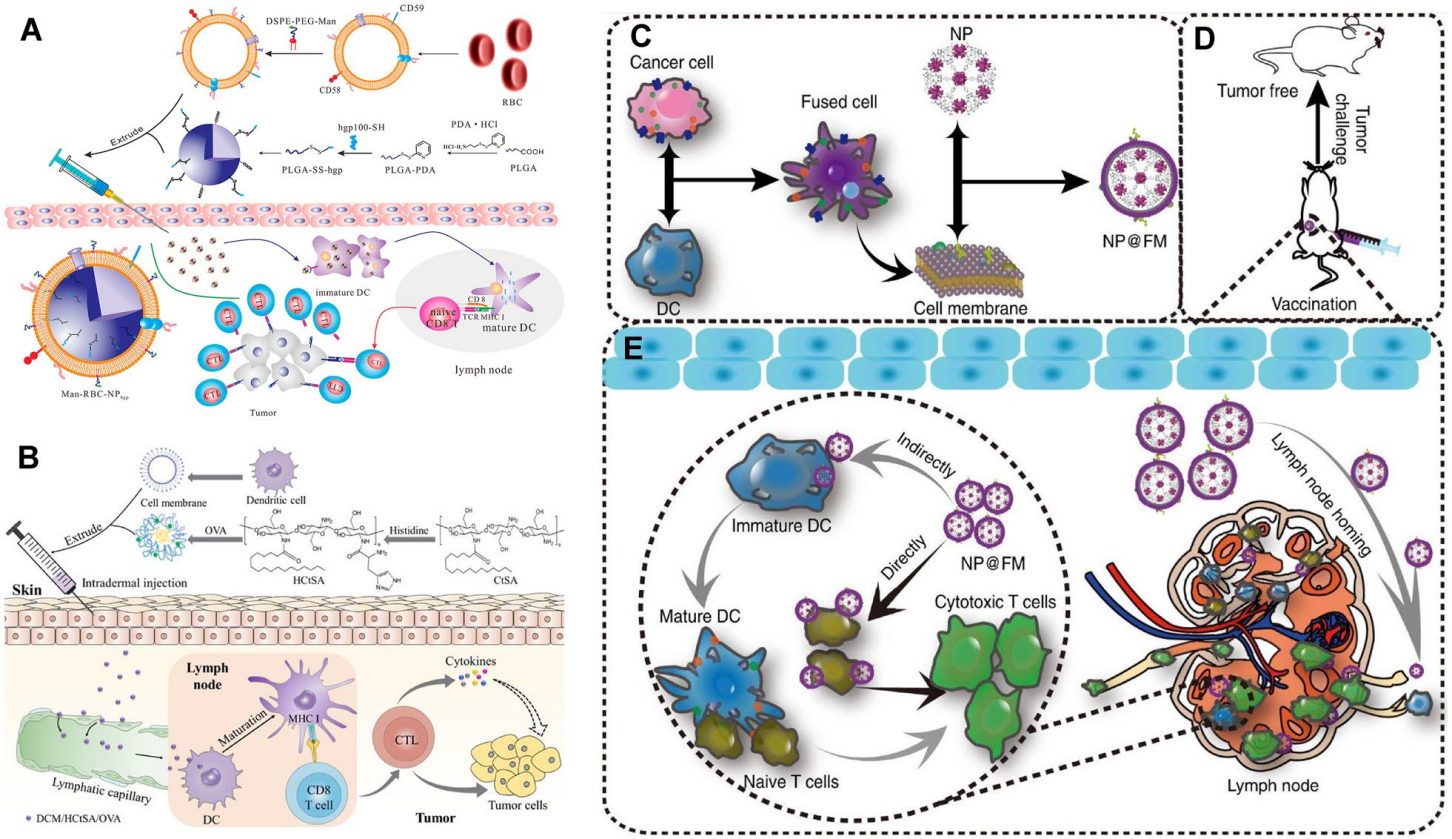
LN-targeting nanoplatforms based on the immune cell membrane. **A** Schematic representation of the preparation and antitumor immune induction of Man-RBC-NPhgp (Adapted with permission from [135]. Copyright © 2015, American Chemical Society) **B** Illustration of the synthesis and anti-tumor mechanism of DCM/HCtSA/OVA. (Adapted with permission from [130]. Copyright © 2020, American Chemical Society) **C** Fabrication process of MOF@FM. **D** MOF@FM inoculation for tumor prevention **E** Mechanisms of MOF@FM targeting LNs (Adapted with permission from [131]. Copyright © 2019, Wen-Long Liu et al., Nature Communications.)

Cell membranes such as erythrocytes, leukocytes, tumor cells, NK cells, MDSCs and platelets have recently been used to camouflage nanoplatforms that can actively interfere with certain phases of the tumor immunity cycle [129]. Red blood cells (RBCs), as the most important blood cells in the body, are able to avoid uptake by macrophage-like cells and systemic clearance due to the abundant “self-markers” such as immunosuppressive protein CD47 expressed on their surface, which enable them to survive for a long time in the bloodstream to better deliver cargo to LNs [129]. Taking advantage of this, Guo et al. inserted the RBC membrane with mannose to actively target DCs in LNs and formed RBC membrane-enveloped PLGA-based nanovaccines (Man-RBC-NPs), which enabled Man-RBC-NPs to combine characteristics of antigen entrapment and stimuli-responsiveness by polymeric NPs with the properties of antigen presentation and adjuvant of RBCs with membrane surface and protein integrity (Fig. 9A) [135]. Benefiting from the ability to actively target DCs, Man-RBC-NPs could efficiently drain to LNs. In the B16F10 melanoma tumor model, Man-RBC-NPs showed good performance in tumor prevention and treatment. More importantly, Man-RBC-NPs did not elicit autoimmune antibodies against RBCs, indicating their safety in the treatment process.

Furthermore, DC membrane-camouflaged nanoplatforms are another kind of commonly used carrier for vaccine delivery. As “professional” APCs, DCs can efficiently home to LNs with the help of chemokines, l-selectin, and integrins expressed on the membrane [136]. Therefore, inspired by the properties of DCs, several nanoplatforms based on DC membranes have been developed to improve the ability to target LNs. For example, faced with the dilemma of inefficient vaccine delivery to LNs and weak cellular immunity due to insufficient antigenic lysosomal escape, which inhibits the strength of the vaccine-induced anti-tumour immune response, Yang et al. synthesized pH-responsive biomimetic vaccines composed of dendritic cell membrane (DCM), histidine-decorated stearic acid-grafted chitosan (HCtSA) and OVA antigen to improve antitumor immunotherapy by targeting LNs and inducing cellular immunity (Fig. 9B) [130]. The homologous targeting of DCM enabled DCM/HCtSA/OVA micelles to target LNs and be captured by DCs in LNs to promote their maturation more efficiently than uncoated DCM based nanovaccines (HCtSA/OVA). More strikingly, DCM/HCtSA/OVA exhibited potent antitumor effects by inducing robust T-cell responses and stimulating the secretion of antitumor related cytokines. Recently, fused cell membrane-coated nanovaccines have attracted more attention. Most cancer vaccines have not been successful in triggering clinically relevant effects and in the absence of exogenous antigens and adoptive cells, Liu et al. have demonstrated that NPs coated with fused membranes composed of membranes from DCs and tumor cells accumulated in LNs more efficiently than either cell membrane type alone [131]. In their study, the MOF@FM nanovaccine was constructed by the metal organic framework (MOF) coated with a reprogrammed cytomembrane from fused cells (FCs) of DC and 4T1 cells (Fig. 9C). By fusing two immunologically related cell types, MOF@FM can not only be effectively retained in draining LNs by the lymphatic homing ability of DCM, but also induce DC-mediated T-cell immune activation without exogenous antigens (Fig. 9D, E). However, although these cell membrane-coated NPs possess good LN homing ability, there are some limitations of this strategy, such as high cost and complexity of production.

#### Exosome-based nanoplatforms

Exosomes are 30–150 nm extracellular vesicles (EVs) and are able to transport a variety of molecules, such as proteins, lipids and nucleic acids as heterogeneous vehicles outside the cell, thus playing a vital role in intercellular communication [137]. Exosomes have been shown to serve as carriers for adjuvants due to their good safety, optimal size, stability in circulation, and ability for targeted delivery [138]. For cancer immunotherapy by tumor antigen vaccination combined with adjuvant, the main challenges include the identification of specific tumor antigens and the effective delivery of antigens as well as adjuvant to APCs. To address this challenge, Morishita et al. developed an efficient exosome-based adjuvant delivery system [132]. SAV-LA-expressing exosomes (SAV-exo) were genetically engineered from murine melanoma B16BL6 cells and SAV-exo were loaded with biotinylated CpG DNA to form CpG-SAV-exo. The results showed that tumor cell-derived exosomes could target LNs efficiently by delivering tumor antigens along with adjuvants to DCs and promoting their migration to LNs. Then, CpG-SAV-exo successfully induced immunostimulatory signals to inhibit tumor growth more significantly than the simple administration of exosomes and CpG DNA in tumor-bearing mice, indicating that the tumor antigen-adjuvant co-delivery system based on exosomes was promising in tumor immunotherapy.

Furthermore, exosomes derived from DCs (DEXs) are also worth of attention as nanovaccine carriers. Zitvogel et al. found that DEXs released from tumor peptide-pulsed DCs presented tumor antigens on the membrane and were able to trigger T-cell immune responses to inhibit tumor growth [139, 140]. They were the first to develop a novel acellular vaccine with exosomes, which was a milestone in the field of exosome-based vaccines. From this point onward, research on exosomes has gradually increased with the development of technology. For example, Phung et al. synthesized a novel bifunctional nanovaccine (EXO-OVA-mAb) using exosomes from ovalbumin (OVA)-pulsed and anti-CTLA-4 antibody-modified DCs, which combined ICB therapy with tumor vaccines [133]. EXO-OVA-mAb not only had an optimal size of targeting LNs but also had a high affinity for LNs by CTLA-4 functionalization of EXO. Therefore, EXO-OVA-mAb effectively drained to LNs through the lymphatic vessels. and triggered robust tumor-specific T-cell responses, while the ratio of effector T cells/regulatory T cells (Tregs) was also increased in C57BL/6 mice bearing B16-OVA. Therefore, EXO-OVA-mAb could produce significant anti-tumor effects in mice, which provided a safe and specific strategy based on DC-derived exosomes for tumor immunotherapy.

#### Neurotransmitter-based nanoplatforms

Polydopamine (PDA) is the polymerized form of dopamine that usually appears in the brain as a neurotransmitter [141]. PDA NPs have been widely used in biomedical fields. The fabrication process of PDA NPs is simple and they not only have high photothermal conversion efficiency but can also load chemical drugs with aromatic rings in the structure [142]. Furthermore, the drug loading and delivery capacity of PDA NPs can also be improved by changing their morphology and structure, such as engraving mesopores inside the particles to form mesoporous polydopamine nanoparticles (MPDA NPs) [134]. For example, Wang et al. developed an MPDA-based nanovaccine PVP-MPDA@R837 for LN-targeting immune activation [134]. Toll-like receptor (TLR) agonists are potent stimulants of the innate immune system and are expected to be adjuvants in anti-tumor immunotherapy. Unfortunately, they are mostly limited by rapid dissemination, leading to ‘wasted inflammation’. In their study, MPDA NPs were loaded with the TLR7 agonist imiquimod (R837) applied for LN-targeting immune activation, and then their surface was coated with the biocompatible polymer polyvinyl pyrrolidone (PVP), which can prevent the interaction between NPs and the stroma and the premature leakage of the drugs. After subcutaneous injection of PVP-MPDA@R837 into the footpad and isolation of the inguinal and popliteal LNs at different time points, they found that PVP-MPDA@R837 accumulated in the ipsilateral popliteal LNs at 24 h and was retained there throughout 96 h, whereas only a small fraction migrated to the ipsilateral inguinal LNs at 48 h, indicating that PVP-MPDA@R837 can efficiently accumulate in draining LNs that maximize drug exposure in the lymphatic system after subcutaneous injection. Furthermore, the antitumor effects were also investigated in a B16F10 murine melanoma tumor model, and the results showed the LNs-targeting immune stimulation using PVP-MPDA@R837 nanoplatform exerted great antitumor effects through thermal ablation and the generation of cytotoxic T lymphocytes for tumor immunotherapy. Therefore, PVP-MPDA@R837, which combines photothermal therapy and immunotherapy, holds great potential in the treatment of tumors.

### Self-assembling nanoplatforms

The emerging and unique design of self-assembled nanostructures can be tailored by the self-assembly behavior of amphiphilic molecules. Self-assembly is a phenomenon observed in amphiphilic molecules, and the different components can self-organize to form stable structures. Self-assembling nanoplatforms can decrease the off-target effects of toxic therapeutics, which benefits LN targeting. Representative self-assembling nanoplatforms for targeting LNs in tumor immunotherapy are illustrated in Table 6.

**Table 6:** Representative self-assembling nanoplatforms for targeting LNs

Class of nanovaccines	Size of nanovaccines	Zeta potential of nanovaccines	Active components	Mechanism of targeting LNs	Anti-tumor effects	Refs.
Albumin/AlbiVax	-	-	Albumin	“Albumin hitchhiking”	Enhanced both innate and adaptive immunity	[143]
hFTN-RFP	11.74 ± 0.8 nm	Negative charge: − 5.69 ± 0.44 mV	REP	Optimal size for LN targeting; Excellent biocompatibility of protein NPs	Inhibited melanoma tumor growth in mice significantly	[144]
PAVX	-	-	JQ1, ICG	Promoting the maturation of DCs	Induced patient-specific immune responses; Blocked PD-L1-dependent immune evasion	[145]
α-Ap-FNP	30 nm	-	α-peptide linked with Ap, CpG	Scavenger receptor class B1 (SR-B1) pathway	Directly elicit potent T-cell mediated immune responses against tumor cells	[146]
DNA-based nanodevice	-	-	Antigen (peptide) TLR agonists (double-stranded RNA (dsRNA) and CpG DNA)	Enhancing APC activation; Reducing nonspecific interactions in vivo	Induced potent antigen-specific CTL responses; Efficient immune-mediated tumor regression	[147]

#### Self-assembling protein nanoplatforms

Endogenous physiological proteins have been widely exploited as vaccine carriers due to their unique physiological properties to allow efficient transport into LNs. Albumin is a 66 kDa endogenous protein. It is the best described example because it can not only efficiently transit to LNs but also penetrate into the LN parenchyma in a stable and safe manner and has low immunogenicity [148–150]. Taking advantage of the “albumin hitchhiking” method, Liu et al. synthesized assembled vaccines in situ based on amphiphiles (amph-vaccines) [151]. In vivo imaging system (IVIS) fluorescence imaging showed that Amph-CpGs exhibited high accumulation in LNs. In another study, considering that subunit vaccines have been studied in several cancer immunotherapy clinical trials with limited efficacy and that nanovaccines can improve efficacy but are rarely translated clinically, Zhu et al. combined Evans blue (EB) derivative (MEB) with molecular vaccines to form the nanovaccine AlbiVax, which could bind to albumin molecules to form albumin/AlbiVax in vivo for efficiently co-delivering adjuvant and peptide Ag into LNs [143]. PET pharmaco-imaging, super-resolution microscopy imaging and light-sheet fluorescence imaging described the transport of vaccines well and confirmed the effective accumulation of vaccines in LNs. Moreover, Albumin/AlbiVax enhanced T-cell responses and significantly inhibited tumor growth. In conclusion, this method has shown a series of advantages: (I) EB-based AlbiVax possesses a good safety; (II) the large-scale production, formula, and quality control of AlbiVax are relatively easy; (III) albumin/AlbiVax possesses a comprehensive and effective delivery mechanism; and (IV) EB-based AlbiVax is expected to be widely applicable to other small molecule therapies.

**Fig. 10:**
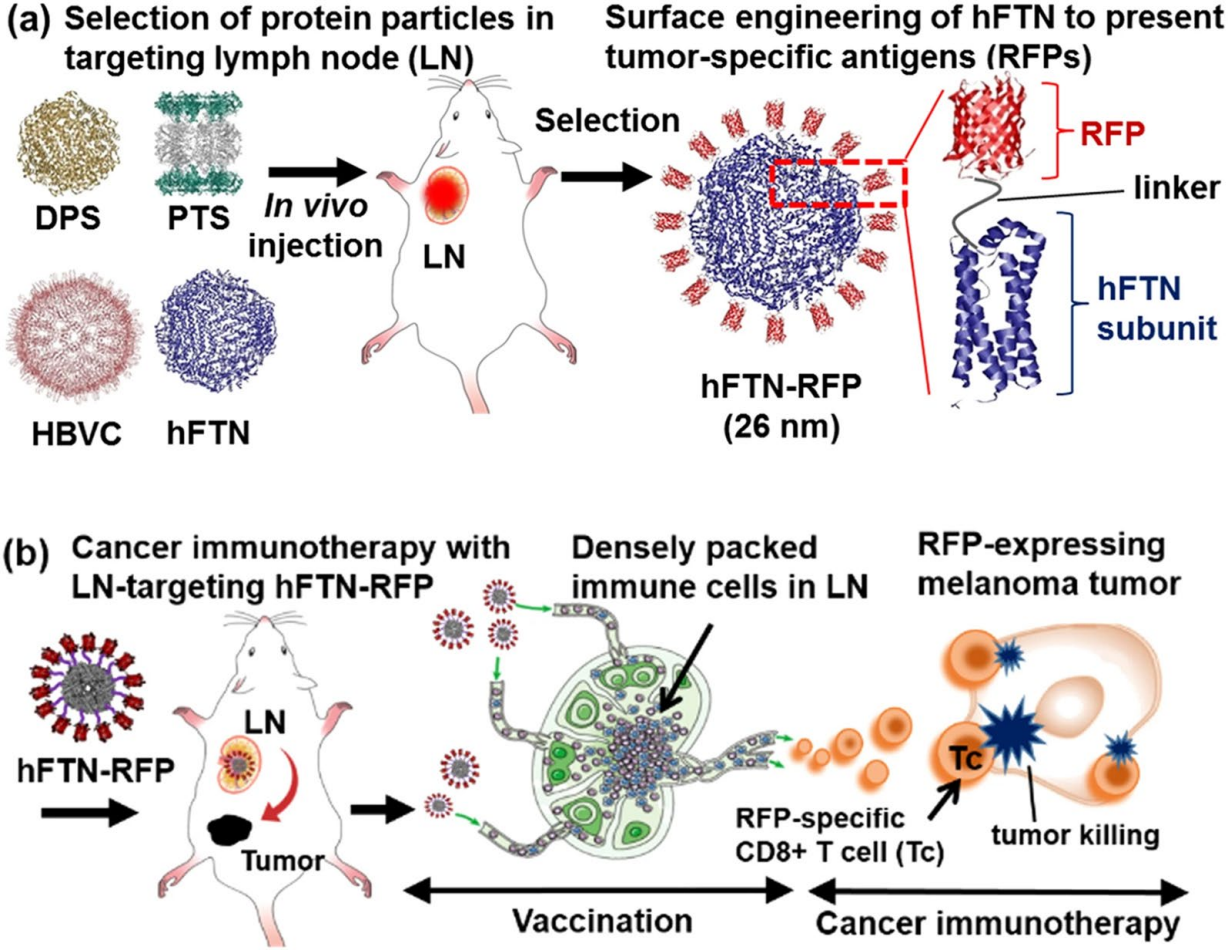
LN targeting-based cancer immunotherapy using self-assembling protein nanoplatforms. Four proteins were evaluated for their LN-targeting ability and hFTN was selected as the LN targeting carrier for the tumor specific antigen (RFP), followed by vaccination using hFTN-RFP via direct LN targeting and finally immunotherapy by RFP-specific CD8^+^ T cells against RFP expressing tumors. (Adapted with permission from [144]. Copyright©2016, Bo-Ram Lee et al., Springer Nature.)

In addition to albumin, Lee et al. also assessed the LN targeting ability of four other protein nanoplatforms with different sizes, shapes and origin [*Escherichia coli* DNA binding protein (DPS, 9.5 ± 1.2 nm, − 5.63 ± 0.33 mV), *Thermoplasma acidophilum* proteasome (PTS, 13.4 ± 2.1 nm, − 2.13 ± 0.27 mV), hepatitis B virus capsid (HBVC, 32.3 ± 1.9 nm, − 7.50 ± 0.43 mV), and human ferritin heavy chain (hFTN, 11.74 ± 0.8 nm, − 5.69 ± 0.44 mV)] [144]. The results showed that hFTN was delivered to the LNs most rapidly and accumulated sufficiently in the LNs. Subsequently, hFTNs were loaded with the model tumor antigen (RFP) to evaluate the anti-tumor effect of hFTN-RFP vaccination. After administration in the mouse model, hFTN-RFP stimulated the production of RFP-specific cytotoxic T cells and significantly inhibited the growth of RFP-expressing melanoma tumors in live mice, indicating its potential as an anti-tumor vaccine carrier in tumor immunotherapy (Fig. 10).

#### Self-assembling peptide nanoplatforms

**Fig. 11:**
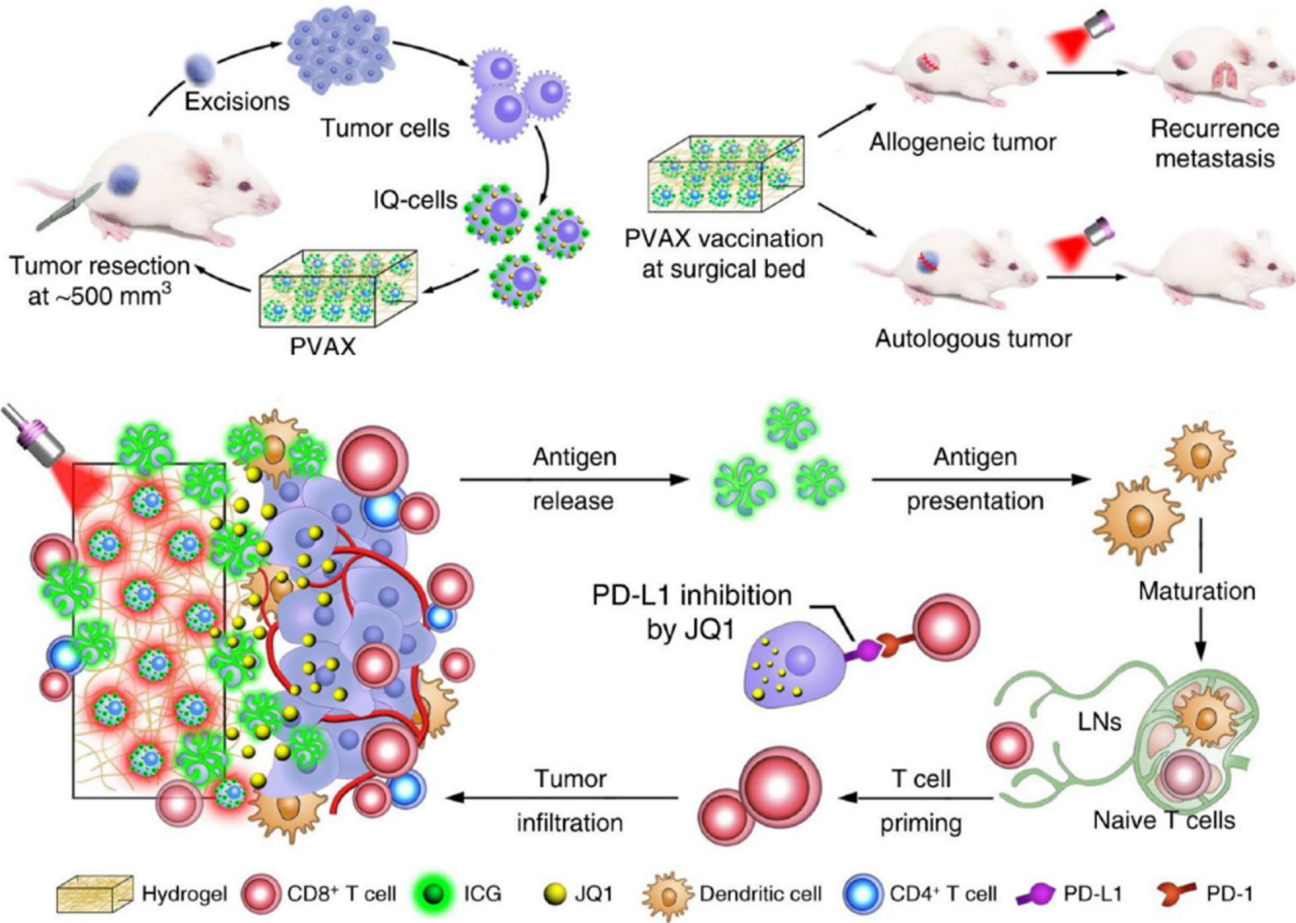
Self-assembling peptide nanoplatforms designed for targeting LNs. Fabrication process and simplified mechanism of PVAX-mediated cancer immunotherapy for preventing post-operative tumor recurrence and metastasis. (Adapted with permission from [145]. Copyright © 2018, Wang et al., Springer Nature.)

Peptides possess programmable self-assembling properties and limited anti-vector immunity, and it has been demonstrated that antigens can be presented by self-assembling peptide fibrils to effectively generate sustained immunity in vivo [145, 152, 153]. Furthermore, Wang et al. also developed personal tumor vaccines (PAVX) with a peptide-based hydrogel matrix, which could overcome the inability of tumor vaccines to simultaneously induce tumor-specific immunity and eliminate immune resistance [145]. Indocyanine green (ICG), which is a photoabsorbent with high photothermal conversion efficiency and BRD4 inhibitor JQ1 co-loaded tumor cells were encapsulated by FK(Fmoc-KCRGDK) peptide hydrogels to form PAVX. The results showed that PAVX could promote the maturation of DCs and enhance the efficiency of their delivery to LNs by 808 nm NIR laser irradiation. In addition, tumor relapse and metastasis were significantly inhibited by NIR light-triggered release of tumor antigen and initiation of tumor-specific immune responses. At the same time, PAVX blocked the PD-L1/PD-1 checkpoint interaction (Fig. 11). Therefore, the kinetics of antigen availability can be regulated by loading active antigens onto nonantigenic peptide scaffolds in physical or chemical ways to promote robust immune responses. Qian et al. have also achieved better LN targeting by directly targeting mature DCs (mDCs) that are abundantly distributed in dLNs [146]. They reported an ultrasmall biocompatible nanovaccine (α-Ap-FNP) whose small size allows for substantial accumulation and targeted delivery of tumor antigenic peptides (Aps) to mDCs via the scavenger receptor class B1 (SR-B1) pathway. Subsequently, further encapsulation of CpG oligodeoxynucleotides on α-Ap-FNPs showed significant results in both prophylactic and therapeutic tumor models.

#### Self-assembling DNA nanoplatforms

DNA can also be used as the carrier of nanovaccines for antigen delivery because of the programmable self-assembly and controlled morphology and size of these molecules, which can form specialized rigid scaffolds that are able to array antigens in a multivalent manner [154, 155]. Taking advantage of the unique properties of DNA scaffolds, Ding et al. developed a tubular DNA origami nanovaccine that can not only protect antigens and adjuvants from protease degradation, but also efficiently mediate their transport to dLNs to induce antigen-specific antitumor immune responses [147]. The DNA robotic nanostructure had a precisely controlled composition. When captured by DCs in dLNs, endosomally localized and pH-responsive nanovaccines are unlocked to mechanically expose antigens and adjuvants at their subcellular functional sites to promote antigen presentation and then invoke robust induction of antigen-specific cytotoxic T lymphocytes (CTLs).

## The application of LN imaging strategies in tumor immunotherapy

The distribution and metabolic process of nanovaccines in vivo can be visualized with the help of LN imaging, which can help us understand the kinetics and efficacy of vaccines. The potential of nanoplatforms has been shown to enhance the quality of LN imaging because of their specific passive targeting ability and efficient co-delivery of cocktail to LNs [156]. In addition, tumor metastasis is one of the landmark biological characteristics of malignant tumor cells, and 90% of tumor-related deaths are caused by metastatic diseases rather than primary tumors, which significantly affects the prognosis of tumor patients [157]. Metastasis of solid tumors in vivo is more likely to occur through the lymphatic system, making LN metastasis one of the most common routes of tumor dissemination [158]. There are two types of LNs that have attracted much attention in tumor diagnosis and treatment, including sentinel lymph nodes (SLNs) and regional lymph nodes (RLNs). SLNs are the first LNs to receive lymphatic drainage from the primary tumor [159], and RLNs are LNs that receive drainage from all areas of the primary tumor [160]. Currently, the main treatment modalities for LN metastasis are surgical resection, local radiotherapy and chemotherapy. Among them, surgical resection is the most widely used method for SLN or RLN dissection. Rapid and accurate localization of SLNs or RLNs and prevention of iatrogenic damage to normal structures during LN dissection remain major challenges [161, 162]. Currently, a series of LN imaging strategies have been developed to address these problems.

### Commonly used imaging modalities

Traditional imaging techniques such as radionuclide imaging, magnetic resonance imaging (MRI), fluorescence imaging (FLI) and photoacoustic imaging (PAI), have been widely used [15]. Radionuclide imaging can label nanovaccines/cells directly or indirectly, and positron emission tomography (PET) is the most commonly used radionuclide imaging method that can be used to directly or indirectly label cells [163]. However, direct labeling usually brings risks such as labeling agent outflow and radioactive exposure, while indirect labeling is usually limited by the high cost of gene editing [164]. MRI possesses the highest spatial resolution of these imaging modalities, and some nanomaterials themselves can be used as contrast agents, such as paramagnetic ion compounds and superparamagnetic/ultrasmall superparamagnetic magnetite complexes [165]. However, MRI is also limited by poor temporal resolution [15]. PAI, as a real-time imaging strategy based on the principle of thermoelastic expansion by absorbing electromagnetic energy, has been widely used for LN imaging because it does not require ionizing radiation and has good safety, but the resolution and sensitivity of PAI are not as good as those of other imaging methods [166]. FLI provides high imaging resolution and tissue penetration depth and mainly provides information at the tissue or cellular level [167]. Conventional NIR imaging utilizes probes that emit within the wavelength range of 700–900 nm (NIR-I window) [168]. With the development of optical imaging technology and probes, optical imaging has been expanded to the NIR-II window (1000–1700 nm), which displays great potential in clinical practice [168–170]. Representative imaging strategies for nanoplatforms-based acellular vaccines and autologous cell-based vaccines are illustrated in Table 7.

**Table 7:** Imaging strategies by nanoplatform-based acellular vaccines and autologous cell-based vaccines

The nanoplatforms used	Imaging mechanism	Imaging modalities	The role of imaging strategies for vaccines	Refs.
5K-HA-HPPS	Loading the core of nanoplatform with DiR-BOA	FLI, PAI	Distinguishing between normal and inflamed LNs and sentinel LNs	[171]
pH-amplified self-illuminating near-infrared NPs	The nanoplatform was covalently conjugated with Luminol and pyrophosphate, a near-infrared fluorescence probe	FLI	Accurate identification of metastatic sentinel LNs	[172]
MEH-PPV@NIR@PEG NPs	Integrating BRET and FRET in an energy transfer relay	FLI	Differentiate between metastatic and benign LNs	[173]
NP-mAb	Labeling of nanoplatforms with the radioisotopes 68 Ga and 177Lu	NIRF, PET, SPECT	Early detection of metastatic SLNs in diverse animal tumor models with small tumor volume	[174]
MR780 NPs	Mannose was connected with near infrared dye IR780 via disulfide bond and then further self-assembled into near infrared nanoprobe with quenched fluorescence	FLI	Significantly improving the sensitivity of in vivo fluorescence imaging	[175]
–	Biocompatible core–shell lead/sulfide quantum dots emitting at ~ 1880 nm;	FLI	Acting as excellent theranostics agents for LN metastasis	[176]
–	Superconducting nanowire single photon detectors for single-photon detection up to 2000 nm"	MRI	Enables precise targeting and non-invasive imaging of metastatic LNs	[177]
–	-	MRI	Overcoming light scattering from biological tissues that limits the penetration depth of high-resolution optical microscopy imaging of living mammals	[178]

### Imaging strategies by nanoplatform-based acellular vaccines

As discussed above, there are usually limitations when only one imaging method is used. To visualize the distribution of vaccines in vivo to accurately target LNs, two or more imaging methods are usually used together to compensate for the shortcomings of a single imaging method. Noninvasive imaging strategies have been extensively investigated for in vivo mapping of SLNs. However, the current imaging strategies fail to accurately assess tumor metastatic status in SLNs with high sensitivity. To discriminate normal LNs (N-LNs) and inflamed LNs (Inf-LNs) from SLNs in breast tumors, Dai et al. developed a CD44 and scavenger receptor class B1 dual-targeting hyaluronic acid nanoparticle (5K-HA-HPPS) based on high-density lipoprotein NPs (HPPS) coupled with HA molecules, and its core was loaded with the NIR/PAI dual-mode imaging contrast agent DiR-BOA [171]. 5K-HA-HPPS could not only rapidly enter SLNs but also target breast tumor cells. The accumulation of 5K-HA-HPPS in LNs could be dynamically monitored by NIR imaging over a long period of time, and the spatial distribution information of 5 K-HA-HPPS in complete LNs could be provided by PAI. To evaluate their LN targeting ability, 5K-HA-HPPSs were injected into the hind footpads of mice, and then wide-field fluorescence imaging was performed. The results showed that 5K-HA-HPPSs migrated rapidly and efficiently to LNs, but this method failed to distinguish Inf-LNs and SLNs. Taking advantage of the high spatial resolution and deep tissue imaging capabilities of PAI, metastatic SLNs could be effectively distinguished from Inf-LNs. After injection of 5K-HA-HPPSs, there were strong PA signals at the periphery of N-LNs and Inf-LNs but weak signals within the LNs, whereas strong PA signals appeared in tumor metastatic SLNs (T-MLNs). Therefore, injection of 5K-HA-HPPS in combination with PAI can effectively identify metastatic SLNs, and thus accurately guide the targeting of nanovaccines. Furthermore, the unique properties of the tumor microenvironment (TME) can also be used to accurately distinguish the target LNs. For instance, utilizing chemiluminescence resonance energy transfer (CRET) and a signal amplification strategy, Wang et al. also visualized metastatic SLNs accurately [172]. In their study, pH-responsive NPs were covalently conjugated with luminol and the near-infrared (NIR) fluorescent probe pyropheophorbide a (PPa) and then formed super-pH-responsive CRET nanosensors (PCNs). PCNs were stable under neutral and alkaline conditions but the disassembly of PCNs was triggered after draining to SLNs and being absorbed by activated macrophages. Therefore, MPO-catalyzed hypochlorous acid in the phagosome could oxidize luminol to emit NIR light by CRET for self-luminescence imaging of T-MLNs. Luminescence imaging of T-MLNs was evaluated in a lymphatic metastasis model that was established by subcutaneous inoculation of 4T1 tumor cells into the right flank of mice. The results showed that significant iridescent luminescence colocalized with the fluorescence signals was detected on the tumor side, whereas no luminescence signal was detected at the normal site. Regardless of whether the tumor was metastatic, the pH-nonresponsive CRET nanosensor (NPCN) was unable to perform luminescence imaging on SLNs. Next, they compared the SLN imaging efficacy in parallel for a series of PCNs with distinct pKa values, including PCN6.9, PCN6.8, PCN6.3, PCN5.3, and NPCN. The results showed that the higher the pHt of PCNs, the stronger the luminescence intensity in SLNs.

In the imaging of intravital organs or cells, conventional fluorescence or confocal microscopy is unable to produce high-resolution and deep-penetration imaging because of the light scattering caused by biological tissues. When it is necessary to image the lymphatic system, particularly when nanovaccines are involved, real-time, intravital and high-magnification imaging especially at the tissue and cellular levels is required [15]. Recently, to solve this dilemma, Dai et al. proposed NIR-IIc confocal microscopy with single-photon detectors and achieved non-invasive cellular-resolution imaging through intact mouse heads and LNs longitudinally [176]. They made a one-photon excitation fluorescence imaging window in the 1700–2000 nm (NIR-IIc) range with 1650 nm excitation, which was by far the longest one-photon excitation and emission for mouse imaging in vivo. In their study, LNs were imaged through intact mouse skin and non-invasive NIR-IIc confocal microscopy imaging at cellular resolution was established in intact LNs in vivo, representing a novel strategy for lymphatic system imaging. In addition, Xiong et al. integrated bioluminescence resonance energy transfer (BRET) and fluorescence resonance energy transfer (FRET) in an energy transfer relay to avoid autofluorescence of living tissues and the scattering and absorption of short-wavelength light in living tissues [173]. They developed MEH-PPV@NIR@PEG nanoparticles (RET1IR) utilizing a nanoprecipitation method. Strong NIR fluorescence signals of RET1IR NPs were detected in the lymphatic networks of mice, indicating the great importance of these NPs for LN mapping and tumor imaging.

**Fig. 12:**
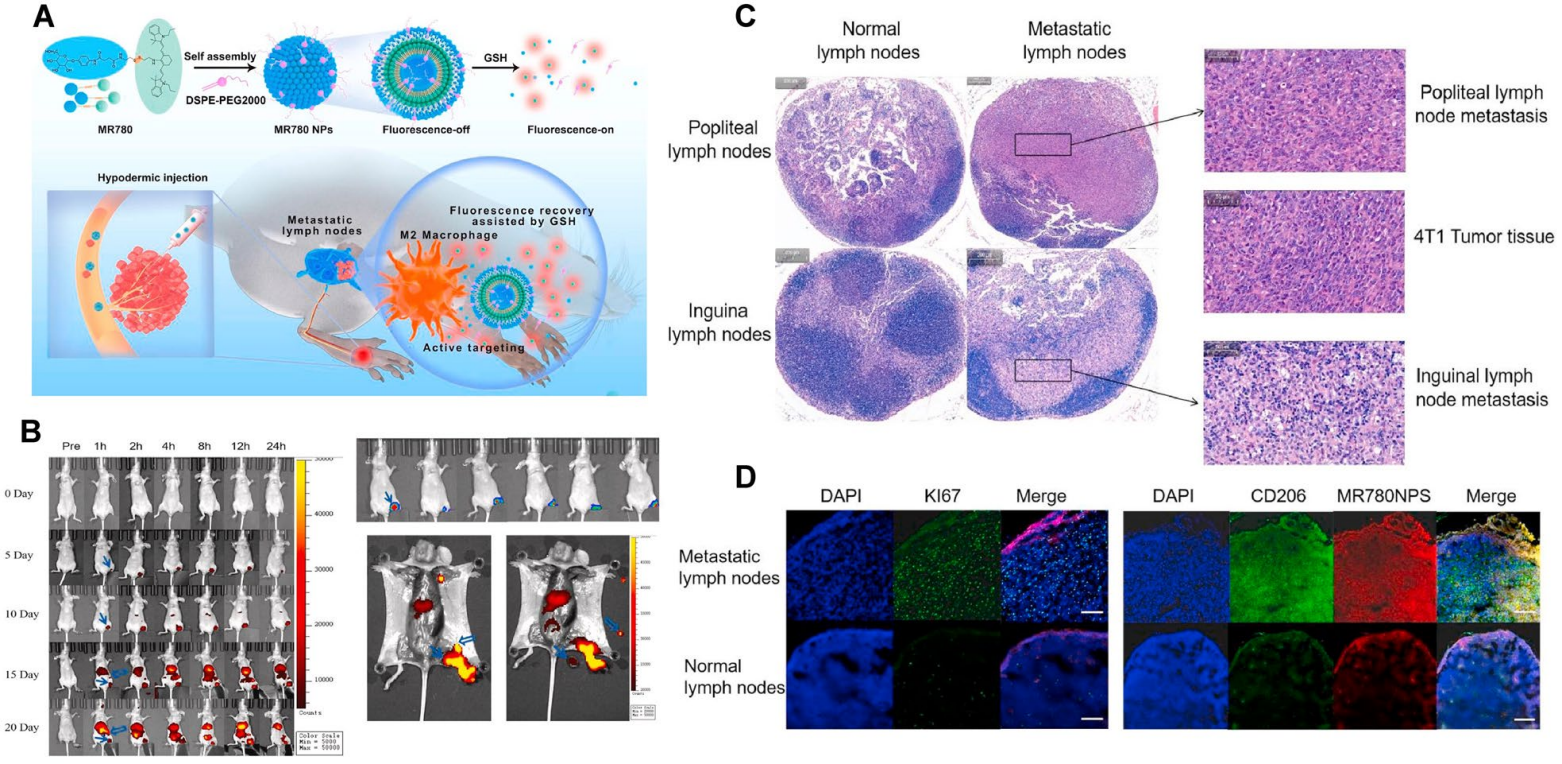
Specific diagnosis of LN micrometastasis via nanoplatform-based acellular vaccines. **A** Schematic diagram of the fabrication process and function of MR780 NPs. **B** Fluorescence imaging of LNs in normal mice and plantar 4T1 tumor-bearing mice every 5 days and fluorescence navigation excision of metastatic LNs. **C** H&E staining of metastatic LNs. (Scale bars: 200 mm for overall view and 50 mm for enlarged view) **D** K167 and CD206 expression in metastatic LNs and MR780 NP aggregation and co-localization in metastatic LNs. (Adapted with permission from [175]. Copyright © 2022 Elsevier Ltd.)

Some LN imaging designs also provide novel lymphatic metastasis targeting strategies for the theranostics of tumors by delivering nanomedicines through the lymphatic system. For instance, Zhang et al. developed NaGdF4:Yb, Tm@NaLuF4 upconversion NPs with PEG and anti-HER2 monoclonal antibody (trastuzumab, Herceptin) (NP-mAb) [174]. NP-mAb could be effectively marked with radioisotopes 68 Ga and 177Lu and formed nanonuclear drug (68 Ga-NP-mAb or 177Lu-NP-mAb). After intratumoral injection into the foot pad, NIRF/PET/SPECT imaging showed that 177Lu-NP-mAb exhibited high accumulation and long residence time in metastatic LNs, indicating that 177Lu-NP-mAb possessed good targeting ability. More importantly, the mouse treated with 177Lu-NP-mAb showed a lower risk of LN metastasis and the primary tumor also decreased. In another study, Zhao et al. connected mannose with the near infrared dye IR780 via a disulfide bond to obtain a mannose-IR780 conjugate (MR780) and then loaded it onto a near infrared nanoprobe (MR780 NPs) with quenched fluorescence, which was mainly used in breast tumors (Fig. 12A) [175]. Mononuclear cells in the surrounding environment are be recruited and differentiate into M2 tumor-associated macrophages (TAMs) when breast tumors have a malignant tendency. Recent studies have shown that a large number of M2 TAMs exist in metastatic LNs [179]. Therefore, MR780 NPs were expected to achieve accurate targeting and noninvasive imaging in their study because mannose can selectively bind to TAM surface CD206 (macrophage mannose receptor, MMR). The results showed that when MR780 NPs specifically bound to CD206 on the surface of TAMs, the abundant glutathione in the microenvironment was able to cut off the disulfide bond and restore fluorescence, indicating the promising potential of LN targeting and imaging of MR780 NPs (Fig. 12C, D).

### Imaging strategies by autologous cell-based vaccines

DC vaccines and T-cell based vaccines can trigger specific anti-tumor responses by modifying autologous cells in vitro and then transferring them to patients [2, 180]. Currently, methods exist for visualizing cell-based vaccines, including (I) direct labeling of cells in vitro; (II) indirect labeling of cells by genetic engineering in vivo; and (III) multimodal imaging [164, 181, 182]. Two types of MRI probes commonly used to label cells are currently iron oxide nanoparticles and GdIII chelates, both of which act as relaxation enhancers for water protons [183, 184]. However, they are not capable of visualizing more than one ensemble of labeled cells in each cell-tracking experiment [185]. In view of this, Carrera et al. proposed a novel type of MRI contrast agent: lanthanide(III) paramagnetic chelates (PARACEST agents), which could track the fates of differently labeled cells that are administered serially [177]. Protons in the pool of exchangeable protons of the agent can transfer saturated magnetization to the bulk water signal upon irradiation at their absorption frequency. Therefore, PARACEST can act as a negative agent by reducing the intensity of the water signal through the transfer of saturated magnetization.

Many studies have reported unique strategies for monitoring the migration of DCs to LNs [164, 186]. Among them, MRI and nuclear imaging are the two most commonly used modalities in mouse models [187]. Furthermore, Figdor et al. have shown that it was feasible to detect very few DCs with detailed anatomical information through magnetic resonance tracking of magnetically labeled cells in vivo [178]. Superparamagnetic iron oxide (SPIO) particles, as the most sensitive markers in existence to label cells for MRI, usually cause safety concerns related to the use of adjunct compounds such as transfection agents in patients [188]. In their study, they took advantage of the fact that immature DCs naturally endocytose SPIO to avoid this phenomenon. They labeled autologous DCs loaded with tumor-derived antigenic peptides with 111In-oxine and SPIO (Endorem) and injected the labeled DCs under the guidance of ultrasound into the LNs to be resected in stage III melanoma patients. The results showed that the properties of DCs in vivo were not influenced by SPIO-labeling, and multimodal imaging described the migration mode of DC vaccines in detail, suggesting that magnetic resonance tracking of magnetically labeled cells is a safe method in clinical practice and that potential of using MRI for tracking therapeutic cells in patients is promising. Regarding T-cell vaccines, gene engineering technology is usually used to make them express luciferase for imaging. For example, Irvine et al. linked liposomes containing the cytokines IL-15 and IL-21 to autologous T cells by maleimide-thiol coupling to enhance the antitumor efficacy of T-cell vaccines [189]. To visualize the behavior of the T-cell vaccine, firefly luciferase (F-luc)-transgenic OT-1T cells were used to visualize the T-cell homing behavior in tumor-bearing mice and click beetle red-luciferase (CBR-luc)-transgenic T cells were used to evaluate T-cell expansion and persistence in a B16F10 melanoma tumor model. In this way, information on T-cell vaccines in vivo can be obtained efficiently. Imaging strategies by nanoplatform-based acellular vaccines and autologous cell-based vaccines are shown in Table 7.

## Conclusion and outlook

With the emergence of nanotechnology, cancer nanovaccines have become one of the most promising therapeutic strategies in the field of cancer immunotherapy, and LNs are the best strategic targets for nanovaccine delivery due to the immense number of phagocytic active resident DCs and their roles in initiating adaptive immune responses. A growing number of studies have demonstrated that targeting nanovaccines to LNs can enhance the final adaptive immune response and promote the transfer of nanoplatforms from the interstitium into lymphatics and from there to LNs, which is a popular and effective strategy for nanovaccine delivery to LNs. Delivery of nanovaccines to LNs usually depends on interstitial transport, and the interstitial pressure and fluid flow rate change with the injection site [190]. Therefore, choosing an appropriate injection site may better promote LN targeting. In addition to traditional intramuscular or subcutaneous injection, immune pathways through the skin containing intradermal, transcutaneous and epidermal vaccination have also been extensively explored. Among them, intradermal vaccination could cause the interstitial pressure to be higher than the lymphatic capillary pressure, which will increase the permeability of the capillary and promote lymphatic drainage, and it is conducive for nanovaccines to target LNs and activate DCs [191]. After administration in a locoregional tissue bed, nanovaccines can transfer from the interstitium into the lymphatics and then drain to LNs or form an antigen depot at the injection site. In the latter method, nanovaccines are captured by APCs in the interstitium and enter LNs through APCs slowly. The size of the nanoplatform is an important factor determining whether the vaccine can enter lymphatic capillaries and subsequently accumulate in LNs. Other determinants include surface modification, shapes and deformability. Despite the rapid development and significant achievements of cancer nanovaccines, there are still several important issues that need to be fully considered before the potential clinical application of nanovaccines, namely:

1. There are currently a few methods that can independently change one variable of the nanoplatforms without changing other variables, making it difficult to generate optimal properties for targeting LNs;
2. The properties of nanoplatforms that promote DC uptake in LNs typically inhibit drainage from the interstitial site of injection;
3. The microenvironment of LNs and lymphatic capillaries will change with pathological conditions such as tumors, inflammation or metabolic diseases and it may be necessary to adjust the targets and strategies for delivering vaccines to LNs based on specific disease backgrounds;
4. Although HEVs support high levels of lymphocyte extravasation from the bloodstream into LNs and other lymphoid tissues, few studies have examined whether nanovaccines can arrive at LNs efficiently via them;
5. Targeting LNs will undoubtedly enhance the induced immune response, but will not completely eliminate solid tumors.

To solve these challenges, the utilization of materials such as scaffolds, hydrogels, and microneedles that are able to improve the delivery of nanoplatforms will undoubtedly continue in the future. Furthermore, it is also worthwhile to explore more ligands specific to target cell types to assist the nanoplatform in better targeting LNs. With the rapid development of instrumentation and imaging techniques, multimodal imaging with different imaging modalities combined together has greatly benefited nanovaccines for cancer immunotherapy, and more combinations of different imaging methods may need to be tested in the future, which does not mean that they are simply combined but that spatiotemporal information can be obtained at different levels. It not only allows for early diagnosis of cancer, but also informs about the characteristics of nanovaccines and the cell populations they affect. Depending on the imaging system, nanoplatforms, cells, or both are labeled with complementary imaging labels. LN imaging has reached a transformative stage, and we need to utilize imaging tools to better optimize nanomedicines for immunotherapy. Considering the number of techniques and agents, there are almost unlimited possibilities for different combinations of imaging modes. Finally, the design of nanoplatforms will continue to be driven by a more detailed understanding of lymphatic biology, mechanisms of vaccine transfer and lymph entry, and LN imaging techniques in the future.

## Abbreviations

LNs: Lymph nodes
APCs: Antigen presentation cells
DOPTA: 1,2-Dioleoyl-3-trimethylammoninum propane
CO: Cholesteryl oleate
DMPC: 1,2-Dimyristoyl-sn-glycero-3-phosphocholine
sHDL-Ag/CpG: DMPC coated with Cho-CpG and Ag
DMPC: 1,2-Dimyristoyl-sn-glycero-3-phosphocholine
Cho-CpG: Cholesterol-modified CpG
cKK-E12: 3,6-Bis (4-(bis(2-hydroxydodecyl) amino) butyl) piperazine-2,5-dione
LPS: Lipopolysaccharide
ssPalm: SS-cleavable and pH-activated lipid-like material
HMs: Hybrid micelles
cGAMP: 2′,5′-3′5′-Cyclic guanosine monophosphate-adenosine monophosphate
PDs/OVA: PAA-PEI600 PDs coated with OVA
PAA-PEI600 PDs: Partially carbonized redox-responsive hyperbranched poly (amido amine) decorated with polyethyleneimine (600 kDa)
Man-CTS-TCL NPs: Natural polymer chitosan coated with mannose whole tumor cell lysates
HA: Hyaluronic acid
MINPs: Magnetic-responsive immunostimulatory nanoagents
SPIO: Superparamagnetic iron oxide
MWNTs: Multiwalled carbon nanotubes
MSN: Mesoporous silica nanoparticle
MPLA: Monophosphoryl lipid A
HCtSA: Histidine-decorated stearic acid-grafted chitosan
MOF: Metal organic framework
FMs: Cytomembrane of the fused cells from DCs and tumor cells
EXO: Exosome
SAV: Streptavidin
MPDA: Mesoporous polydopamine
PVP: Polymer polyvinyl pyrrolidone
hFTN: Human ferritin heavy chain
REP: Red fluorescence protein
ICG: Indocyanine green
TLR: Toll-like receptor
